# Study on the persistence of Zika virus (ZIKV) in body fluids of patients with ZIKV infection in Brazil

**DOI:** 10.1186/s12879-018-2965-4

**Published:** 2018-01-22

**Authors:** Guilherme Amaral Calvet, Edna Oliveira Kara, Silvana Pereira Giozza, Camila Helena Aguiar Bôtto-Menezes, Philippe Gaillard, Rafael Freitas de Oliveira Franca, Marcus Vinicius Guimarães de Lacerda, Marcia da Costa Castilho, Patrícia Brasil, Patrícia Carvalho de Sequeira, Maeve Brito de Mello, Ximena Pamela Diaz Bermudez, Kayvon Modjarrad, Robyn Meurant, Sihem Landoulsi, Adele Schwartz Benzaken, Ana Maria Bispo de Filippis, Nathalie Jeanne Nicole Broutet, for the ZIKABRA Study Team, Adele Schwartz Benzaken, Ana Izabel Costa de Menezes, Ana Maria Bispo de Filippis, André Luiz de Abreu, Anna Thorson, Armando Menezes Neto, Camila Helena Aguiar Bôtto-Menezes, Carlos Alexandre Antunes Brito, Daniele Simões, Edna Oliveira Kara, Guilherme Amaral Calvet, Kayvon Modjarrad, Lydie Trautmann, Maeve Brito de Mello, Marcia da Costa Castilho, Marcus Vinicius Guimarães de Lacerda, Massimo Ghidinelli, Morgane Rolland, Nathalie Jeanne Nicole Broutet, Ndema Habib, Patrícia Brasil, Patrícia Carvalho de Sequeira, Philippe Gaillard, Pierre Formenty, Rafael Freitas de Oliveira França, Rasmi Thomas, Robyn Meurant, Ronaldo de Jesus, Sihem Landoulsi, Silvana Pereira Giozza, Ximena Pamela Diaz Bermudez

**Affiliations:** 1 Acute Febrile Illnesses Laboratory, Evandro Chagas National Institute of Infectious Diseases, Oswaldo Cruz Foundation, Rio de Janeiro, Rio de Janeiro Brazil; 2 World Health Organization, Geneva, Switzerland; 3 Department of STI, AIDS and Viral Hepatitis, Secretary for Health Surveillance, Ministry of Health Brazil, Brasilia, Brazil; 4 Tropical Medicine Foundation Doctor Heitor Vieira Dourado (FMT-HVD), Manaus, Amazonas Brazil; 5 Aggeu Magalhães Research Center, Oswaldo Cruz Foundation, Recife, Pernambuco Brazil; 6 Flavivirus Laboratory, Oswaldo Cruz Institute/Oswaldo Cruz Foundation, Rio de Janeiro, Brazil; 7 Department of Communicable Diseases and Health Analysis, Pan American Health Organization/World Health Organization, Washington DC, USA; 8 Public Health Department, University of Brasilia, Pan American Health Organization/World Health Organization, Brasilia, Brazil; 9 Walter Reed Army Institute of Research, Silver Spring, USA

**Keywords:** Arbovirus, Flavivirus, Zika virus, Body fluids, Persistence, Rt-Pcr, Emerging infectious diseases

## Abstract

**Background:**

Zika virus **(**ZIKV) has been identified in several body fluids of infected individuals. In most cases, it remained detected in blood from few days to 1 week after the onset of symptoms, and can persist longer in urine and in semen. ZIKV infection can have dramatic consequences such as microcephaly and Guillain-Barré syndrome. ZIKV sexual transmission has been documented. A better understanding of ZIKV presence and persistence across biologic compartments is needed to devise rational measures to prevent its transmission.

**Methods:**

This observational cohort study will recruit non-pregnant participants aged 18 years and above with confirmed ZIKV infection [positive reverse transcriptase-polymerase chain reaction (RT-PCR) test in blood and/or urine]: symptomatic men and women in ZIKV infection acute phase, and their symptomatic or asymptomatic household/sexual infected contacts. Specimens of blood, urine, semen, vaginal secretion/menstrual blood, rectal swab, oral fluids, tears, sweat, urine and breast milk (if applicable) will be collected at pre-established intervals and tested for ZIKV RNA presence by RT-PCR, other co-infection (dengue, Chikungunya, HIV, hepatitis B and C, syphilis), antibody response (including immunoglobulins M and G), plaque reduction neutralization test (if simultaneously positive for ZIKV and dengue), and ZIKV culture and RNA sequencing. Data on socio-demographic characteristics and comorbidities will be collected in parallel. Participants will be followed up for 12 months.

**Discussion:**

This prolonged longitudinal follow-up of ZIKV infected persons with regular biologic testing and data collection will offer a unique opportunity to investigate the presence and persistence of ZIKV in various biologic compartments, their clinical and immunological correlates as well as the possibility of ZIKV reactivation/reinfection over time. This valuable information will substantially contribute to the body of knowledge on ZIKV infection and serve as a base for the development of more effective recommendation on the prevention of ZIKV transmission.

**Trial registration:**

NCT03106714. Registration Date: April, 7, 2017

**Electronic supplementary material:**

The online version of this article (10.1186/s12879-018-2965-4) contains supplementary material, which is available to authorized users.

## Background

### Epidemiology

ZIKV is a mosquito-borne arbovirus in the family *Flaviviridae*, genus *Flavivirus* whose first isolation occurred in 1947 from a rhesus monkey in the Zika forest of Uganda [1]. The genus includes more than 70 viruses comprising important pathogens such as dengue, yellow fever, Japanese encephalitis and West Nile virus [2]. All flaviviruses include in their cycle a vertebrate host and insect vectors and many flaviviruses are clinically important as they elicit central nervous system diseases in infected individuals [3]. The primary mode of ZIKV transmission is through mosquito bites. The virus is almost exclusively transmitted by the *Aedes aegypti* mosquito but has been isolated from several other species of the genus Aedes such as *Aedes albopictus*, *Aedes africanus* and *Aedes luteocephalus* [4]. Any country where competent vectors are present bears risk for ZIKV outbreaks. In addition to the ongoing outbreak started in 2015 in South America, ZIKV has previously caused outbreaks in the Yap Islands, French Polynesia, New Caledonia, Cook Island and Easter Island [5]. The mosquito has a predilection for indoor environments which might explain its distribution reflecting patterns of human migration and urbanization [6]. Between 01/01/2007 and 02/06/2016 Zika was documented in 60 countries and territories of which 46 were experiencing their first outbreak since 2015 [7].

### Clinical manifestations of ZIKV infection

The most common symptoms of ZIKV infection are rash, fever, arthralgia, myalgia and conjunctivitis [8]. Recently a study in Rio de Janeiro, Brazil, identified maculopapular rash (97%) and pruritus (79%) as the two most common symptoms among confirmed cases [9]. Most of the patients with ZIKV infection have mild disease, but severe neurologic complications have been described in patients in French Polynesia and during the current outbreak in Brazil [10, 11].

ZIKV infection has become a major public health concern due to suspected causal relationship between ZIKV and microcephaly, amongst other neurological manifestations. The Pan American Health Organization/World Health Organization (PAHO/WHO) issued an alert in November 2015 in response to a large increase in the number of cases of microcephaly notified by the Brazil International Health Regulations National Focal Point. The country, which had its first autochthonous case confirmed in May 2015, and by November 2015 had spread to 15 states, experienced a staggering increase in rates of microcephaly in live births from 5.7/100,000 in 2010 to 99.7/100,000 by November 2015 [12]. This led the World Health Organization to declare the current outbreak as a Public Health Emergency of International Concern on 1 February 2016 [13].

Microcephaly is a rare neurological condition where babies are born with a small head in comparison to other babies of the same sex and age. The symptoms accompanying microcephaly depend on the level of disease severity and vary from mild to life threatening. There is no specific treatment for this condition. In addition to genetic factors, microcephaly can also be associated with other so-called STORCH infections, (i.e. syphilis, toxoplasmosis, rubella, cytomegalovirus and herpes simplex virus), maternal exposure to toxic substances and malnutrition during pregnancy [14].

### Sexual transmission of Zika virus

ZIKV is the first flavivirus to be known to be sexually transmittable; current evidence suggests that sexual transmission has been increasing.

Sexual transmission of ZIKV was first suggested by Foy et al. in 2008 [15]. The authors described two cases that, after returning to their home in Colorado, US from Senegal, developed symptoms of ZIKV infection. Case 1 had sexual intercourse with his wife 1 day after his return and she developed symptoms of ZIKV infection 4 days later. She had no history of traveling outside the US during the previous year.

On 2 February 2016 the United States Centers for Disease Control and Prevention announced the first documented case of sexual transmission of ZIKV between two men through anal sex [16]. Suspicion of transmission through oral sex was raised in a case report published in April 2016 [17]. The case had sexual contact (vaginal intercourse, with no condom and no ejaculation, and oral sex with ejaculation) with her ZIKV infected symptomatic partner. Sexual transmission has been reported to occur up to 41 days after symptom onset in the index case [18].

By June/2016 ten countries have reported cases of sexually transmitted ZIKV (Argentina, Chile, Peru, US, France, Italy, Portugal, New Zealand, Canada and Germany) [7]. All index cases reported were male and presented symptoms consistent with ZIKV infection after travelling to areas of high ZIKV activity. Transmission of the infection to their sexual partners (male or female) could have occurred before, during or after symptoms onset [19]. None of secondary cases had other risk factors apart from having sexual activities with male partners who have been to areas of ongoing high ZIKV transmission activity.

### Asymptomatic ZIKV infection

Around 80% of individuals infected with ZIKV remain asymptomatic [4]. A likely man-to-woman sexual transmission of ZIKV between two asymptomatic persons was first reported in June 2016 [20]. Until this date all reported sexual transmissions implicated a male index case with symptoms of ZIKV infection. This finding may play a more important role than expected in the dynamic of ZIKV transmission. This is of concern mainly for pregnant women or those considering pregnancy and needs to be further investigated to inform counselling and recommendations.

### What is known about the presence of the Zika virus in body fluids

#### Semen

ZIKV was first isolated in semen during a ZIKV outbreak in French Polynesia in December 2013 [21]. The case was a man in Tahiti whom, after having two episodes of symptoms compatible with ZIKV infection, sought treatment for hematospermia 2 weeks after recovering from the second episode. His semen was found positive for ZIKV through RT-PCR testing. In 2016, up to June, two studies looking at the presence of ZIKV in semen were published. The first, published in March, reported a case whose viral load in the semen, found more than 2 weeks after symptom onset, was 100,000 times that of the patient’s blood [22]. In the second study, published in May, British researchers documented the case of a 68 year-old man returning to UK from Cook Islands in 2014 [23]. ZIKV particles were detected in his semen (RT-PCR) 62 days after his symptoms began. The virus, however, was not cultured and further specimens were not collected and analyzed. The length of time during which the virus can persist in semen remains unknown.

Important questions need to be addressed regarding sexual transmission of ZIKV and its duration in semen. Among them are whether there are differences in persistence of ZIKV between the semen of men with symptomatic and asymptomatic ZIKV infection.

#### Saliva

Similar to other flaviviruses for which detection in saliva has been previously documented, ZIKV particles have also been detected in saliva [24, 25].

In 2013, during the French Polynesian outbreak, researchers collected a saliva specimen to replace an unrealizable blood sample from a 1 year old child. Because ZIKV RNA particles were detected, they further investigated the presence of the virus in the saliva, during the same outbreak, as an alternative method for detection of the virus during the acute phase of the infection. In comparison to blood, the ability to detect ZIKV particle through RT-PCR in saliva was found to be higher. However, because detection could be negative in saliva while positive in blood, the latter could not be replaced [26].

ZIKV has been detected in the saliva of another case who spent 2 weeks in the Dominican Republic in January 2016 and was admitted to a hospital in Venice, Italy, with symptoms compatible to ZIKV infection [25]. Specimens demonstrated persistent shedding of ZIKV RNA in saliva and urine for up to 29 days after symptom onset, while viral RNA was detectable in plasma up to day 10 after symptom onset.

#### Menstrual blood, vaginal and rectal secretions

Menstrual blood, vaginal and rectal secretions are associated with sexual activities and for this reason will be collected from participants in this study. This will contribute to improve knowledge on the presence of the ZIKV in these fluids so interventions and management practices can be devised. By the time this protocol was produced no report has been published on the detection of the virus in these body fluids.

### Other body fluids

#### Breast milk

The inclusion of breast milk as one of the body fluids to be studied is due to its importance as a possible mode of vertical transmission. Questions have been raised as to whether transmission of ZIKV can occur during breastfeeding. To date, no case has been documented on transmission of ZIKV from mother to baby through breast milk. Breastfeeding has been previously suggested as a possible vertical route of transmission for other viruses belonging to the *Flaviviridae* family such as dengue and West Nile viruses [27, 28]. In July 2015 a case of ZIKV infection was reported in New Caledonia related to a 27 year old febrile mother whom after 2 days of delivering a healthy baby, at 37 week gestation, developed a maculopapular rash. Both mother and baby had blood samples collected and the mother had also breast milk samples taken. Infective particles of ZIKV were found only in the mother’s specimens (35,000 RNA copies per mL in serum and 850,000 RNA copies per mL in the breast milk) through RT-PCR [29].

In the current context of ZIKV transmission, in light of recent evidence, and due to the benefit of breast milk for mothers and babies, the World Health Organization recommendations on breastfeeding have not changed. Infants should start breastfeeding within 1 hour of birth and continue for 6 months exclusively and up to 2 years of age or beyond, after the introduction of complementary food [30]. Further research, however, is needed on the ZIKV transmissibility through breast milk, and its persistence.

### Sweat and tears

Studies on the detection of the ZIKV in sweat and tears have not been published up to June 2016. This is an opportunity to increase generalizable knowledge and reduce uncertainty about ZIKV infection and, according to the results, to contribute to public health recommendations and actions.

### Urine

The isolation of ZIKV in cell culture from urine was first described in 2014 in Canada [31]. The study reported the case of a Canadian traveler returning from Thailand who was initially diagnosed with dengue fever as result of preliminary laboratory investigations. Atypical dengue serological results, however, led the investigators to a reconsideration of infection by another flavivirus.

In February 2016 ZIKV was also isolated in Brazil where urine and saliva samples from 9 patients in Zika acute phase symptoms were inoculated in Vero cell culture resulting in one isolate returned from urine (and one from saliva) [32].

It was suggested that renal excretion of ZIKV can start around 4 days after symptom onset [33]. ZIKV RNA, however, has been found in urine 20 days after it was no longer detected in serum [34]. The urine has shown to yield positive results through PCR, in a patient with Guillain-Barré Syndrome after 21 days of the neurological symptoms onset [35].

### Gingival crevicular fluid

Gingival crevicular fluid (GCF), a serum transudate or inflammatory exudate present in the gingival crevice surrounding teeth, and saliva are identified as distinct body fluids. The constituents of GCF originate from serum, gingival tissues, and from both bacterial and host response cells. Whole saliva and GCF can be distinguished by the fact that the IgG content of the GCF is several times greater than that of saliva.

As this is a non-invasive means of diagnosis, monitoring of viral markers and assessing the host response in infectious disease, and because saliva collection is less technique-sensitive than GCF, collection the latter will also be performed in the participants. No reports on the presence of ZIKV in GCF have been published so far.

### Study hypothesis and objectives

Study Hypothesis*:* ZIKV can be shed in human body fluids long after the time of the acute infection. Persistence of ZIKV in different body fluids may vary due to the influence of circulating specific ZIKV IgM and IgG, as well as host and environmental factors.

#### Primary objectives

1. To assess the presence and duration of ZIKV and related markers (ZIKV specific RNA, antigen, antibodies, T cell response and innate immunity) in blood, semen, saliva, crevicular fluid, urine, vaginal secretions, menstrual blood, rectal swab, tears, sweat and breast milk of infected individuals who present to clinics during acute illness and convalescence and in their infected symptomatic and asymptomatic household/sexual contacts.
2. To relate these parameters to host immunity over time and across different body fluids, to socio-demography factors as well as other host and environmental factors.

#### Secondary objectives

1. To analyse the presence and duration of ZIKV in bodily fluids amongst male and female adults aged 18 years and above over a 12 months period.
2. To analyse the persistence of other markers (ZIKV RNA, IgM, IgG, antigen) of ZIKV infection in bodily fluids amongst male and female adults aged 18 years and above over a 12 months period.
3. To explore potential relation between host and environmental factors as well as co-infections - dengue, chikungunya, HIV, syphilis, HBV, HCV, Oropouche virus (Bunyavirus), Mayaro virus and Yellow Fever (check immunization status) - on the duration of the persistence of the virus in body fluids. HIV, syphilis, HBV and HCV tests will be provided by the National AIDS control programme. The algorithms used by the programme for testing and treatment will be used.
4. To assess the proportion of asymptomatic infections among household/sexual contacts to infected cases.
5. To assess the possibility of reinfection or reactivation of the ZIKV cases over the study period.
6. To describe concordance between RT-PCR test results, neutralizing antibodies, virus isolation, antigen results, and specific ZIKV antibody responses in these fluids.

## Methods/design

### Study design

This is a prospective observational cohort study. After signing a screening consent, male and female potential study participants presenting rash compatible with ZIKV infection in collaborating clinics will be tested for the presence of ZIKV in blood and urine. The following day, after providing informed consent for enrolment, those found to be RT-PCR positive for ZIKV infection, fulfilling all inclusion criteria will be enrolled and subject of regular collection of body fluids over a period of 12 months according to a pre-established schedule. Household/sexual contacts will be screened at the reference centers where the index case was diagnosed or if preferable at their household, and recruited following the same criteria. The standard care will be provided to all participants.

### Index case – Screening

- **Screening inclusion criteria**
  - ○ Being a man or woman aged 18 years and above
  - ○ Presenting rash compatible with ZIKV infection
  - ○ Having given consent to provide blood and urine for RT-PCR testing and to be enrolled if the tests return positive, including coming back to all follow-up visits and provide body fluid according to the testing schedule.
  - ○ Permanence for 12 months at a place that allows sample collection for this period
- **Screening exclusion criteria**
  - ○ Being under 18 years of age
  - ○ For women, being pregnant
  - ○ Presenting a condition that would not allow reliable informed consent (e.g. alcohol abuse and substance misuse)
  - ○ Lacking mental capacity to give informed consent to participation

### Index case - enrolment

- **Enrolment inclusion criteria**
  - ○ Presenting RT-PCR test positive for ZIKV in blood and/or urine specimens collected during screening
  - ○ Having given consent to provide all body fluid collection, as appropriate, according to the testing schedule
- **Enrolment exclusion criteria**
  - ○ Presenting RT-PCR test negative for ZIKV in blood and/or urine specimens collected during screening

### Household contacts (or sexual contacts if index case lives alone) - screening

- **Screening inclusion criteria**
  - ○ Being a man or woman aged 18 years and above
  - ○ Having given consent to provide blood and urine for RT-PCR testing and to be enrolled if the tests return positive, including coming back to all follow-up visits and provide body fluid according to the testing schedule
- **Screening exclusion criteria** ***-*** As per index case

### Household contacts (or sexual contacts if index case lives alone) - enrolment

- **Enrolment inclusion criteria-** As per index case
- **Enrolment exclusion criteria** ***-*** As per index case

### Procedures

#### Study sites

The following criteria were used for the selection of the study sites:

- High population density
- High circulation of ZIKV
- Strong community health network
- Laboratory facilities able to perform viral culture, ZIKV antigen assays, RT-PCR, IgM/IgG, neutralizing antibodies test (specific for ZIKV, dengue and chikungunya) and genetic sequencing of ZIKV.

As per criteria above the sites to take part in the study are: Rio de Janeiro (RJ); Manaus (AM) and Recife (PE). All are capital of states in Brazil.

#### Screening health facilities

Potential participants (index case) will be identified while seeking clinical care at reference center ambulatory, primary health centers and 24 h emergency units. Explanation on each of these services is provided on the next section of the protocol.

#### Enrolment and specimen collection health facilities

The settings for enrolment and specimen collection will include the reference center ambulatory. Potential participants screened at primary health centers and 24 h emergency units will be referred to the reference center ambulatory. Household of the index case will be referred as appropriate.

#### Testing health facilities

The testing of specimens will be performed at the Fundação Oswaldo Cruz (Fiocruz) Rio de Janeiro, RJ and Recife, PE; and Fundação de Medicina Tropical Dr. Heitor Vieira Dourado (FMT-HVD) in Manaus, AM. Additional supplemental testing will be performed at Walter Reed Army Institute of Research, Bethesda, MD, USA.

### Study participants

Participants will comprise men and women aged 18 years and over with diagnosis of ZIKV infection. They will include symptomatic cases diagnosed at the study collaborating clinics (index cases) and their asymptomatic or symptomatic household/sexual contacts.

### Participant recruitment and enrolment

#### A. Participant recruitment

##### Index cases

The identification of symptomatic subjects will take place in the collaborating clinics: Fiocruz ambulatory (Rio de Janeiro and Recife), FMT-HVD ambulatory (Manaus), primary health centers and 24-h emergency units located in the areas of the study intervention.

At each of these screening sites, a triage system will be in place to direct subjects meeting study inclusion criteria for consideration for enrolment.

1. Fiocruz

Fiocruz is a center of reference for patients with rash and fever. Individuals who meet the study inclusion criteria seen at Fiocruz and who live nearby the center, due to the logistic of home visit by the study nurses, will be invited to partake.

2. Primary health center- family clinics

In Brazil, Family Clinics are Health Clinic Units where the main focus is the prevention and early diagnosis of diseases as well as health promotion. The clinics comprise multi-disciplinary groups of professionals including doctors, dentists, nurses, community health workers, amongst others, who provide services such as individual appointments, home visits, immunization, antenatal care, cancer screening, sexually transmitted disease testing and prevention, smoking cessation, among other services tailored to the patient’s needs [36].

3. 24-h emergency units (Unidade de pronto Atendimento, UPAs)

UPAs are health services between Emergency services and Health Basic Units known as UBS (Unidade Básica de Saúde). UPAs are open 24 h a day, 7 days a week and their main objective is to relieve the work done by Emergency Departments to where more urgent and severe cases are referred to. Patients attending the UPAs can be treated at the unit or, depending on their clinical manifestations, be either referred to a hospital or to one of the Health Basic Units [37].

Both Family Clinics and UPAs already work in collaboration with Fiocruz in ongoing research studies. Health professionals working in both services will be informed about the study, particularly the eligibility criteria, and asked to identify and refer suspect cases of ZIKV infection to Fiocruz for further evaluation and enrolment as appropriate.

1. Municipal Health Center-MHC (Centro Municipal de Saúde)

Municipal Health Centers operate similarly to the Family Clinics described above but with a higher level of complexity and greater number of services offered to the population of the area it covers.

A study nurse will be based in the MHC in the community to be identified by the study team. This MHC will be located in an area of the city with high population density and known cases of ZIKV infection. In this MHC suspect cases of ZIKV infection will be referred to the study nurse by health professionals who will be informed about the study and inclusion/exclusion criteria. The study nurse will assess their eligibility, obtain the informed consent, apply a screening questionnaire and perform specimen collection.

##### Household/sexual contacts

Permission from individuals diagnosed with ZIKV infection (index case), and who accepted to take part in the study, will be requested to approach their household contacts/sexual contacts and invite them to participate. This permission will be documented in the informed consent applied by the study nurse. Index cases will be asked to inform their household contacts/sexual partners about the study and explain the possibility of them being screened as well for participating. If applicable, household contacts/sexual contact will either come directly to the clinic for screening or be visited at home by the study nurse who will explain the study and follow the same procedures undertaken for the index case. If feasible, invitation to partake will take place in the same day the index case received their positive result for ZIKV infection. Should this be not possible, a visit for the following day (nurse to household or household/sexual contacts to the clinic) will be organized or as soon as possible thereafter, but not later than 2 days after enrolment of the corresponding index case. Household/sexual contacts will include infected (symptomatic or asymptomatic) individuals aged 18 years and above. Individuals who agree to take part will follow the same procedures applied to the index. This will include screening informed consent (at the clinic or the household), questionnaire completion (at the clinic or the household) and first body fluid collection (at the clinic only). The screening informed consent will be given for:

1. Completion of a short baseline socio-demographic questionnaire;
2. Drawing blood;
3. Providing urine specimen;

Individuals will receive the testing results within 24–48 h of the specimen collection. If results are positive for urine and/or blood the participants of the screening will be asked to give consent for their participation in the full study (enrolment consent).

Should a household/sexual contact be absent during the nurse visit, flexibility will be provided in terms of appointment bookings, taking into account that specimens need to be collected as soon as possible after the index case was diagnosed.

#### B. Participant enrolment

Cases will be enrolled at places where study nurses will be based: Fiocruz ambulatory, FMT-DVD ambulatory or MHC. As described earlier, MHC will only recruit participants screened within its structure, while Fiocruz ambulatory will recruit participants screened within its structure and also receive referral from the primary health centers and 24-h emergency units. The contact details of the participant will be collected and their eligibility assessed by the study nurse that is, whether they meet the inclusion criteria and doesn’t meet any of the exclusion criteria. After the eligibility checks, the study will be explained in detail to the participants, including what the collection of each body fluid will entail and the frequency at which body fluid specimens will be collected. After making sure the participants have no further questions about the procedures, they will be asked to sign an enrolment informed consent. Illiterate participants will have the consent form read to them in the presence of a witness, who will sign to verify the accurate reading of the form and agreement of the participant.

This enrolment informed consent will be given for:

1. Completion of a more detailed questionnaire (enrolment questionnaire) and follow up questionnaires
2. Providing blood, urine, saliva, oral fluid (saliva and gingival crevicular fluid), sweat and tear specimens, rectal swab and breast milk specimens (if lactating).
3. Providing semen specimens (for male participants) or vaginal and menstrual blood specimens (for female participants).
4. Storage of part of the specimens provided and the information associated with them. The specimens will be stored in the participant laboratories and used for the following purposes: further research studies, development of drugs and/or vaccines. Patients will be consulted before each new study which will be reviewed by an appropriate ethics committee.
  A bio-repository, linked to the ZIKABRA protocol, will be created aiming at the possibility of use in future investigations. Therefore, after processing and acquisition of the results, the remaining biological material will be stored with the intention of carrying out different analyzes than the current protocol in one or more studies in the future. According to the Brazilian resolutions, the term of validity of this type of bio-repository can be authorized for up to 10 years, being possible renewals authorized by the relevant ethics committees (CEP/Conep) through an examination of justification and report presented by the researcher. For each new research, it is necessary to apply a new Informed Consent form (or, when duly justified, to obtain approval of the dispensation of the Term by the Committee) for the use of stored biological material that was previously collected. This information is provided in the informed consent. If consent is not given for future use, the specimens will be discarded at the end of the current study according to national guidelines.

After the consent is signed and dated, each participant will be assigned a unique identifier number and a card with this number will be provided to the participant.

Each collaborating center will be responsible for keeping the signed consent forms securely for both index case and their household/sexual contacts.

The clinical management of patients is not a part of this research protocol. It will be at the discretion of the medical consultant and carried out according to standard of care at the treating site where recruitment occurred.

### Sampling and sample size calculation

ZIKV is an emerging pathogen about which little information is known. This is a prospective observational cohort study, whose prediction of the exact sample size required cannot be prospectively determined. However, considering the scarcity of existing information on viral shedding, even a few participants would contribute to the body of evidence on the topic and lead to improved public health recommendations.

A multistage cluster sampling will be used to identify potential participants for inclusion and follow-up in the persistence cohort. All consecutive individuals RT-PCR positive for ZIKV in urine and/or blood identified during the screening process will be included in the cohort. In addition, all adult household/sexual contacts (symptomatic or asymptomatic) of these clinical cases, will be offered participation, and to be tested and included in the cohort if found RT-PCR positive for ZIKV in blood and/or urine.

The following is the rationale for estimating the sample size needed for body fluids other than blood, when we hypothetically estimate the prevalence of persistence will be 50% after 12 months. The below sample size calculations refer to estimating a population proportion at a certain point (or period) in time.

Using a population size (N) of 1,000,000 for finite population correction factor, and hypothesized rate of ZIKV persistence in at least one body fluid that is near 50% by the end of 12 months post-ZIKV infection, with a two-sided 95% confidence interval width of 10% (or margin of error (ME) of ±5%), a sample size of 385 males and 385 females who are either positive for ZIKV in urine or in blood at baseline will be needed.

The potential introduced design effect of the different clinic facilities used in the consecutive recruitment of study participants, will be explored both in stratified analyses by clinic, and in addition clinic will also be run as an independent variable in multivariate models.

To account for the design/clustering effect at household level, we applied design effect DE of 1.5 assuming a cluster (family) size of 3 ZIKV-infected participants and an assumed an intra-cluster correlation (ICC) of 0.25, resulting into a sample size of 576 males and 576 females. We further assume that 10% of participants, index cases and household/sexual contacts, will be lost to follow up, by the end of the first year and hence the final sample size will be 634 males and 634 females. We rounded this number up to 650 males and 650 females. The sample size is tentatively estimated, given that there is little information on ZIKV persistence rates in bodily fluids, available in literature. The final sample size of 650 males and females is estimated for evaluation of the primary outcome which is the overall ZIKV persistence rate by 12 months (taking into consideration a design / clustering effect and loss to follow up). Since the role of different risk factors is not yet well known, the subgroup analyses will be done in an exploratory way and the effect of different factors on the primary outcome will be further explored in multivariable analysis models.

### Admission procedure

#### Index case

- **Screening visit (visit 0)**

- As a first step during the screening visit, the study nurse will explain the study to potential participants. Pictograms depicting the collection of different body fluids will be used for facilitating the communication between them.
- After ensuring the participant understood the information provided and that all their questions have been answered, an initial informed consent will be signed. The informed consent will state that the specimens will be collected during screening and after enrolment, when applicable, and that participation in the full study will only occur should a positive result for ZIKV infection be found in the patient’s blood and/or urine. A short questionnaire (Screening questionnaire) with basic socio-demographic data will be applied. This is the initial part of a more detailed questionnaire that will be completed if tests yield positive results and the individual continues in the study.
- Specimen collection will be completed in a secure, private space in the study clinic using appropriate infection control precautions. Blood and urine will be tested using RT-PCR.
- The participant will receive explanation about the next steps. These steps will depend on whether the ZIKV RT-PCR testing in blood or urine turn out to be positive or negative.
- The participant will also receive information on the communication occurring with his/her household contacts and/or sexual partners. The index case will be informed of the necessity to visit the household, or receiving the household members/sexual partner at the clinic for explanation about the study, informed consent, short questionnaire, and collection of specimens at the clinic at the same or following day of the result delivery.
- The laboratory results for RT-PCR of blood and urine of the index case should be available within 24–48 h after specimens arrived at the laboratory, regardless of the day of the week.
- An appointment will be made with the index case for the following day for delivering of the test results (visit 1).
- At each study site, participants will receive a financial amount to cover the costs of their transport and a meal. This will happen at each study visit. Individuals not willing to attend the collaborating clinic will be offered the alternative of being visited at home by a study nurse at their convenience.

b) **Enrolment visit (Visit 1):** Laboratory results delivery - the day results for blood and urine specimens are available. The test result will be delivered to the participant by the study nurse at the enrollment site.

- Negative RT-PCR for ZIKV in blood and urine

For individuals whose ZIKV RT-PCR tests performed on urine and blood returned negative, management will be done according to regular procedures and they will be referred back to the initial clinic where they were seen in the first place.

- Positive RT-PCR for ZIKV in blood or urine

Individuals whose RT-PCR tests performed on blood or urine are positive will be considered as index cases. The next steps will be the following:

- ○ The nurse will explain in details what the full enrolment in the study entails and ask for an informed consent for the index case to continue in the full study.
- ○ A detailed questionnaire will be applied (Enrolment questionnaire).
- ○ The nurse will proceed with the specimen collection of all body fluids discussed previously with the index case, including new blood and urine specimens.
- ○ The specimen collection schedule will proceed according to the time frame and intervals shown in Table 1.
- ○ Specimens collected will be noted on forms.
- ○ The nurse will also discuss with the index case the invitation of the household contacts/sexual partners to the study.

**Table 1:** Time frame for collection of specimens

Time	Recruitment visit index case	Recruitment visit household member	Sample collection		
Visit 0	Sample collection	–	Blood and urine		
Visit 1 – inclusion visit	Visit 0 + 2 days				
	RT-PCR Results				
	-RT-PCR positive: individuals start follow up	Invitation to attend the clinic or household visit by the nurse (Blood and urine)			
	-RT-PCR negative: individuals are not included in the study				O
Visit 2	Visit 1 + 2 days	Visit 1 + 3 days			T
Visit 3	Visit 1 + 4 days	Visit 1 + 5 days			H
Visit 4	Visit 1 + 10 days	Visit 1 + 11 days	B		E
Visit 5	Visit 1 + 20 days	Visit 1 + 21 days	L		R
Visit 6	Visit 1 + 30 days	Visit 1 + 30 days	O	+	
Visit 7	Visit 1 + 60 days	Visit 1 + 60 days	O		F
Visit 8	Visit 1 + 90 days	Visit 1 + 90 days	D		L
Visit 9	Visit 1 + 120 days	Visit 1 + 120 days			U
Visit 10	Visit 1 + 150 days	Visit 1 + 150 days			I
Visit 11	Visit 1 + 180 days	Visit 1 + 180 days			D
Visit 12	Visit 1 + 210 days	Visit 1 + 210 days			S
Visit 13	Visit 1 + 240 days	Visit 1 + 240 days			
Visit 14	Visit 1 + 270 days	Visit 1 + 270 days			
Visit 15	Visit 1 + 300 days	Visit 1 + 300 days			
Visit 16	Visit 1 + 330 days	Visit 1 + 330 days			
Visit 17	Visit 1 + 360 days	Visit 1 + 360 days			

#### Household/sexual member

- **Screening visit:** The first visit of the household/sexual member to the collaborating clinic OR visit of the study nurse to the household.

- The visit will start with a detailed explanation on the study following the same steps applied to the index case’s first visit. Once the prospective participant has no questions or doubts about the study aims and what their involvement entails, an initial consent form will be signed (Screening informed consent).
- The screening questionnaire will be applied in a private room.
- Irrespective of where the explanation about the study, informed consent and questionnaire were administered (household or clinic), all specimens will be collected in a private room at the clinic.
- Only blood and urine specimens will be collected during the screening visit.

b) **Enrolment visit**

- If blood or urine specimens are positive, the process for specimen collection will follow the same steps described to the index case.
- All body fluids will be sent to the reference laboratory on the same day of collection.
- In addition to RT-PCR, specimens from asymptomatic contacts will be tested for antigen and IgM at day 1 and 20. This will be an opportunity to diagnose asymptomatic household/sexual contacts by IgM seroconversion.

### Follow-up procedures

Participants with positive RT-PCR in either blood or urine will be asked to return for a follow up visit according to the study pre-established schedule (Table 1) which will have been discussed when the informed consent was sought. There will be one day flexibility from visit 2 in case for some reason the participant is not able to attend the clinic for specimen collection. Individuals who consent and have yielded a positive result for ZIKV infection will be followed for 12 months in order to evaluate for persistence of virus, reactivation and reinfection.

Menstruating women will be asked to provide a specimen as close to the first day of their period as possible, and a second specimen before the end of the period. Menstrual blood sampling may or may not be done at the same time as other body fluid testing. If the woman’s period does not coincide with other specimens’ collection times, she will be asked to attend for specimen collection on different days as appropriate. This will be explained by the study nurse at the time of the recruitment.

A study card ID check will be produced for each participant taking part in the study and the information with the correspondent identity for each ID will be locked in a safe file. In case of ID loss by the participant, the locked in ID may be accessed to allow verification of the participant’s identity.

Contact information will be reviewed at each visit. Text messages will be sent by the administration assistant prior to the appointments as reminders. If applicable, calls will be made to participants who have missed their appointments, and flexibility will be provided in terms of appointment bookings and any needs for additional information. In addition, community healthcare workers from the areas where the study will take place will be involved in the study to be in contact with the participants.

### Counselling

Appropriately trained study nurses will provide counselling on ZIKV infection to index cases, their household and sexual contacts. This will include the following information:

- Ways of transmission of ZIKV infection: via mosquito bites, mother to her fetus, unprotected sexual contact with an infected person;
- Safer sex/risk reduction;
- Strategies to prevent unintended pregnancy;
- Pregnancy planning: Couples will be informed according to the latest evidence based by the time of the counselling (the counsellors will be updated each time recommendations are changing);
- Positive men whose partners are pregnant will be counselled on how to prevent sexual transmission of ZIKV through sex;
- Women will be offered pregnancy test and if found to be pregnant they will not be included in the study and will be referred to antenatal care;
- Participants will also receive counselling for conditions identified during the specimen testing. Individuals in need of specific health service will be referred as appropriate by the study doctor.

### Criteria for discontinuation of a participant

The prospective data collection from a participant including specimen collection will be discontinued after one year of the first specimen collection.

If male participants are unable to provide a semen specimen at two consecutive visits, they will be referred to specialized urological care if necessary. Additional eligible participants may be recruited to replace these cases.

A participant who cannot be contacted after 3 attempts (phone contact and home visit) for two consecutive visits or who does not present to the study clinic for follow-up visit for two consecutive visits will be considered as lost to follow-up.

After careful evaluation of risks and benefits of the research for pregnant women, and considering the frequency of collection of body fluids, pregnancy was considered as one of the exclusion criteria of the study, aiming at not provoking additional stress to pregnant women due to collections of body fluids and frequent visits to the study centers. According to this criterion of exclusion, and aiming above all the benefit for pregnant woman, women that become pregnant during the course of the study will have their participation discontinued for the same reasons.

They will be, however, promptly referred to existing specialized units for the care of pregnant women with Zika, where they will receive adequate care for their special needs and be followed up to delivery. All information and exams related to this patient and collected during the study period will be shared with the referred unit. In addition there is also the possibility of invitation to participate in ongoing cohort studies of pregnant women infected with ZIKV. This is available in all cities where the study is being conducted.

### Laboratory and other investigations

All laboratory results will be filled into a form marked with the participant’s study ID and kept in confidential storage for entering of follow-up results.

Depending on the tests to be performed, different tubes will be used for blood collection:

- ○ To obtain serum, whole blood will be collected through venipuncture in plain vacutainer tubes (or equivalent) allowed to clot and serum separated after centrifugation. Venous blood specimen will be drawn to enable analysis of Zika IgM and IgG antibodies in serum and for the ZIKV Ag assay.
- ○ To obtain plasma whole blood will be collected through venipuncture in a vacutainer tube containing EDTA, allowed to stand or centrifuge to separate plasma.
- ○ For cytokine determination the correct anticoagulant should be heparin.

### Screening

**Visit 0**: blood and urine

- Rapid pregnancy test
- Blood: 2 tubes of 5 mL without anticoagulant (SST tube) for ZIKV RT-PCR and biochemistry and 1 tube of 5 mL EDTA for haematology
- Urine: 10 to 50 ml in universal urine analysis tube for ZIKV RT-PCR

### Enrolled patients

**Visit 1 and following visits** – for RT-PCR positive patients (enrolled). The steps below will be followed for specimen collection of specific body fluids:

Pregnancy test: rapid urine test.

Diabetes screening: blood drop – glucometer tests

- Blood
- Visit 1, 4, 6–17
  - ○ Brazil: HIV, syphilis, hepatitis B and C; RT-PCR for ZIKV, Dengue, Chikungunya. Immune response: IgG, IgM, ZIKV antigen, serological screen for exanthematic virus, Oropouche virus (Bunyavirus) and Mayaro virus, Yellow Fever (check immunization status);
  - ○ Walter Reed: PRNT, micro neutralization, flow based neutralization, T cells assays, cross reactions;
  - ○ 2 × 10 mL SST tubes should optimally be collected;
  - ○ 1 × 10 mL heparin tube.
- Visit 2, 3, 5
  - ○ 1 × 10 mL SST

- Urine (for ZIKV RT-PCR; antigen; HCG; virus isolation)

- 20–50 mL of urine will be collected in a screw top urine container
- 1 aliquot of 2 mL to be processed immediately for RT-PCR
- 1 aliquot of 2 mL of urine will be frozen at − 70 °C for antigen
- 3 aliquots of 2 mL will be frozen for future studies

Considering that for the following fluids is not possible to predict the final volume that will be available after collecting, it is important to consider that is necessary to have at least 500 μl for RT-PCR and serology (if applicable) and an additional 500 μl to be kept frozen at − 70 °C for future studies.

For the following specimen types, one fresh aliquot should be processed for RT-PCR for ZIKV and 2 aliquots should be stored frozen at − 70 °C for Ag test and culture:

b) Oral fluids (saliva, crevicular fluid)
c) Semen

- Ejaculate
- Collected in a screw top urine container

d) Sweat
e) Tears
f) Vaginal secretion
g) Menstrual blood
h) Breast milk
i) Rectal secretion

Note: Participants will be asked for consent their specimens to be used for future research. Specimens of consenting individuals will be kept at − 70 °C. Specimens that test positive by RT-PCR will also be processed for culture/viral isolation.

WHO will provide guidance on assay selection. Laboratories from different sites will be using the same assays, reagents and equipment.

### Confirmation of recent ZIKV infection

Laboratory confirmation of recent ZIKV infection will comprise:

- Presence of ZIKV RNA or antigen in serum or other specimens (e.g. oral fluid, tissues, urine, whole blood); or
- When the patient is found to have nucleic acid specific for ZIKV as well as chikungunya or dengue, confirmatory testing of serum will be required. ZIKV infection will be confirmed when: IgM antibody against ZIKV positive and PRNT90 for ZIKV with titre ≥20 and ZIKV PRNT90 titre ratio ≥ 4 compared to other flaviviruses; and exclusion of other flaviviruses.
- Virus culture will be performed in order to determine whether a given body fluid specimen has live virus. The results of the virus isolation will be reported to the correspondent study nurse.

### Provision of results to participants

All laboratory results will be filled into a form marked with the participant’s study ID and kept in confidential storage for entering of follow up results.

### Genotyping/selected full-length sequencing

Additional genomic analysis will be performed on a subset of positive samples by full-length sequencing methods. Whole genome sequencing and cognate viral load measures will be explored. Confirmatory follow-on procedures including real-time quantitative PCR will be performed as necessary, preferably with commercial reagents or alternatively those laboratory developed assays adapted from the literature. Viruses may be obtained by co-culturing with activated peripheral blood mononuclear cells (PBMCs), if other molecular methods for virus sequence determination fail.

### Lymphocyte Polyfunctional studies and Immunophenotyping

Assays for cellular and humoral immune responses will be measured with the specimens stored during the study and will be determined based on the level of technology at the time. CD4+ and CD8+ T cells, B cells and NK cellular responses may be evaluated by a variety of assays including multi-parameter flow cytometry, intracellular cytokine staining and ELISPOT assays. Cytokine levels and inflammatory biomarkers may be measured by sandwich-ELISA method using validated commercial cytokine kits or alternative methods such as Luminex or other multiplexed platforms. Further advances in immunologic assays will occur and may be utilized. Immunophenotyping according to multiple lineage and differentiation markers may be carried out depending on the availability and salience of cutting edge assays. Additional cell types may also be evaluated by flow cytometry. Additionally, single cell immunophenotyping analysis, single cell sorting, antibody Fc-effector profiling and functionality assays and antibody cloning may also be performed.

### Host genetic testing

Investigators will perform targeted analyses of genetic polymorphisms within host genes reported to influence the rates of pathogen acquisition and/or disease severity. These genes may function pathogen host restriction, and innate and adaptive immune system responses. Host restriction genes of interest include but are not limited to entry receptors and post-entry factors. Targeted genes of the innate immune system will include, but not limited to, killer immunoglobulin receptors (KIR) and cognate Class 1 ligands; dendritic cell-specific intercellular adhesion molecule-3-grabbing non-integrin (DC-SIGN); and toll-like receptors (TLR). Those involved in humoral and cell-mediated immune responses will include Fcγ receptors, capable of binding diverse immunoglobulin isotypes; and the alleles comprising Class I HLA-A, -B, and -C loci, respectively. HLA typing procedures that will be used in this study have yet to be validated for clinical use, but represent essential research tools for the analyses of adaptive immune responses across multiple types of infections. HLA typing and other genetic testing data will be only collected from those who have provided specific consent in a separate genetic testing consent form. Data will be unlinked from personal identifiers, and reported in aggregate for the populations studied.

### Transcriptomics

Isolated cross sectional identification of genomic expression is fraught with a lack of reproducibility due to patient heterogeneity, latent confounding and non-biological variation. Importantly, the quantitative approach developed for analyzing associations between within-patient gene changes and clinical outcomes conducted longitudinally should provide more robust predictions of outcomes than single measurements of gene expression controlling for a bulk of the intra-patient confounding and heterogeneity. The challenge is how to accurately interrogate and associate early longitudinal gene expression measured at multiple time points with long term clinical trajectories captured by a constellation of clinical variables. We seek to capitalize on the longitudinal structure within an individual, combining bioinformatics and statistical tools to elucidate pathway dynamics from the gene expression data. For host transcriptomics, approximately a small amount of blood will be collected into commercial blood RNA tubes, processed at the hospital, and then frozen for transport to the laboratory. Total RNA will be extracted with commercial kits and subjected to quality control. Specimens meeting quality control criteria will be further processed for use on a human gene expression microarray. Extracted RNA will be reverse transcribed and amplified using kits. Arrays will be scanned raw images will be processed using proprietary software. The gene lists generated will be formatted and subjected to network or biological pathway analysis. Networks will be further analyzed for relevance to the particular biological system and clinical setting.

### Study instruments

The following instruments have been produced to provide measure of the variables of interest:

### Questionnaires

Some of the key variables will be measured through questionnaires administered by the study nurse. Three types of questionnaires will be used to collect information: Screening questionnaire (to be completed during the screening visit – see Additional file 1); Enrolment questionnaire (to be completed after RT-PCR for ZIKV positive results are delivered – see Additional files 2 and 3); and Follow up questionnaires (see Additional files 4 and 5).

- **Screening questionnaire**: completion will be performed at the first visit by the study nurse. The same questionnaire will be applied to males and females and includes questions on social demographic data.
- **Enrolment questionnaire**: this is a gender specific questionnaire divided by sections as follows:

- Socio demographics data (more detailed in comparison to the screening questionnaire)
- Sexual and Reproductive health history
- Epidemiological data
- Comorbidities
- Vaccines
- Health assistance
- Sexual activity

c) **Follow up questionnaire**: this is also a gender specific questionnaire which will be applied at the follow up visits by the study nurses. The questionnaire will collect information on the convalescent phase of the ZIKV infection and will comprise questions on health and wellbeing and sexual activity.

**Consultation forms** (see Additional files 6 and 7).

These are medical forms to be completed by the study consultant in infectious disease during medical consultations. Information collected will include:

- Signs and symptoms: onset and end of each symptom in both phases, acute and convalescent.
- Physical examination: general, circulatory, respiratory, neurological and rheumatological.

### Data quality assurance

Quality checks will be built into the data management system used by the study team and quality control checks of critical data points will be employed to ensure standardization and validity of the data collected. In case of invalid answer an alert will be issued providing the possible answers for that specific question. A manual as well as training of persons who will be involved in the completion of the questionnaire will be provided.

Data queries will be generated and conveyed to each participating site. Ownership of the data entered into the central database will remain with the site that contributed it.

The database will be installed in a dedicated server for the management of the data in Java©. The server will allow authorised members of the study team to enter online data which will provide great flexibility as data entry will be possible at the point where the data is being collected. At the same time, consistency of answers will be verified during the process of questionnaire completion. This will be possible through any internet navigator. In addition to the security measures for accessing the system there will be automatic backups.

Nurses will use ultrabook (tablets) for entering the questionnaire responses collected during the visits to the index case household or at the collaborating clinics. Access to the database will be given to those individuals who will be entering and analyzing the data.

Members of the team with access to the database will be trained before receiving access to the productive server. Likewise only trained interviewers will be used for the interviews and specimen collection. The topics included in the questionnaires include asking for information that may be perceived as sensitive and efforts will be made to ensure a private setting and calm environment to assure internal validity of the data collected.

Handling and treatment of biological sampling and specimen will follow standard operating procedures. Specimens will be handled at appropriate laboratories following relevant protocols.

Study staff will review data collected and entered for quality and consistency. Data quality reports will be developed and generated weekly with feedback to study staff.

### Data management

- WHO in collaboration with the Ministry of Health data management team will design an electronic web based database, compliant with good clinical practice. Information collected from the questionnaires and clinical forms will be entered from each site and monitored by a member of WHO team. For data capture the platform to be used will be OpenClinica and analyses will be performed with SAS. All people who collect and analyse the data will be given access to the database. Laboratory results will be imported from an electronic management system utilized by Public Laboratories in Brazil.
- Data entry, validation, and query resolution will run in parallel with recruitment.
- No patient-identifying information will leave the country and will be managed by the collaborating clinics. Each clinic will capture their own data and export to a central unit for analysis which will be performed by a statistician contracted by the study. Individual records and the key linking the participant code number will be kept secure, accessible only to the local study team under the supervision of the study PI.
- Interview data will be organized in databases stored on secure local servers within each study site and it will be backed-up regularly. Collected data will be directly recorded on computers or tablets to minimize data recording and entry errors and minimize delays in data availability. If paper forms must be used, interview responses will be entered into the database either daily or as a group at the close of data collection; and 5% of entered forms will be re-checked to identify any problems with data entry accuracy that must be addressed.
- Electronic equipment and files will be kept password-protected.
- Paper forms and electronic devices will be kept locked when not in use.
- All patient identifiers will be removed from the dataset before analysis and replaced with a unique participant code that can be linked back to individuals via a master key at a centralized secure server and database.
- Paper interview forms, if used, will be destroyed within five years, according to the Brazilian National Regulations (Resolution 466/2012), after all data are entered and verified. However, questionnaires administered by the study nurses are meant to be completed electronically.
- Laboratory staff will identify specimens only by the labelled study ID, and will not have access to any personally identifying information.
- Study nurses will provide laboratory results only in person and only to participants who (1) return for a follow-up study visit; (2) present their study ID card or confirm their identity and study ID number; and (3) agree to receive their own results. Individual results will not be shared with anyone other than the study participant. Results will be presented according to a script.

### Data analysis plan

- Descriptive analysis of the study participants will be performed by a statistician located at the country where the study is undertaken.
- WHO technical assistance may be provided as needed for these and any additional analyses of the study data. Any further statistical analysis will be conducted using adequate statistical program packages.
- Viral persistence time will be measured in days from time after symptom onset or identification of the infection in asymptomatic participants.
- Measures of interest are:
  - ○ Maximum duration of tissue culture-positive results for ZIKV for each of the body fluids specimen types;
  - ○ Maximum duration of other markers of ZIKV infection tested for each of the body fluids specimen types tested for these markers;
  - ○ Viral load as determined by cycle threshold (CT) value and/or international units per mL (IU/mL) for each specimen with positive RT-PCR result;
  - ○ Comparison of ZIKV genetic sequences between participants and from specimens collected over time from the same participant;
  - ○ Rate of co-morbidity with HIV, syphilis, dengue and chikungunya viruses;
  - ○ Correlation between positive RT-PCR and culture tests for each body fluid type.
- Viral persistence in a body fluid at a certain period in time, will be analyzed in relation to host factors such as age, sex, co-morbidity including HIV, syphilis, dengue virus and chikungunya virus and its clinical characteristics, severity of the symptoms, as well as presence of IgM and IgG antibodies in serum, and environmental factors.
- Participant information on sexual history will be analyzed for frequency and type of sex (protected/unprotected).
- Statistical methods:
  - ○ The baseline characteristics for the entire sample of recruited participants will be described;
  - ○ Frequencies and percentages will be reported for categorical variables. For quantitative normally distributed variables, the number of subjects, means and standard errors will be provided, while medians, interquartile range, minima and maxima will be reported for skewed non-normal quantitative variables;
  - ○ The analysis for the study outcomes will primarily be based on the eligible participants who tested positive for ZIKV in blood or urine at baseline;
  - ○ The Kaplan-Meier (K-M) survival analysis (non-parametric methods), will be used to analyze time to event outcomes (utilizing both right-censored and interval-censored techniques), K-M survival curves will be plotted and the Cox Proportional Hazards semi-parametric model to adjust for potential confounders/independent variables. Parametric survival models would also be explored;
  - ○ For the categorical (binary) repeated outcomes models that take into account correlation between outcomes collected over time will be considered. This would include GEE models, where relevant;
  - ○ Two-sided tests and 5% significance levels will be used and 95% confidence intervals for all relevant parameters. SAS statistical packages will be used for the statistical analyses;
  - ○ Open-ended questions will be listed and coded for meaningful comparisons of their distribution.

### Independent data monitoring committee

An Independent Data Monitoring Committee (IDMC) will be established by the Study management team and sponsors with the aim to ensure that the interests of patients taking part in the study are protected and that the scientific soundness of the study is kept from onset to completion. The IDMC shall be independent of the study and free of significant conflicts of interest.

The IDMC shall review the interim data at four intervals namely: after visit 8 (90 days), after visit 11 (180 days), after visit 14 (270 days) after visit 17 (360 days) for the study progress towards its goals and its integrity (compliance with the protocol, study sites performance and recruitment).

The size of the IDMC will range from 3 to 5 members of which two designated positions should be a chair who will lead the deliberations and sign the official documents and a statistician to minimize subjective judgment.

The IDMC will be supported by the study coordinator, Statistician and/or Data Management Team, as needed. They may be required to present reports at the IDMC meeting, but have no voting rights and may be excluded from parts of the meeting.

The IDMC will report to the study (principal investigator and sponsors) or Trial/study management team and sponsor and notify safety concerns as soon as possible.

### Ethical issues

#### Ethical considerations

##### Study population, recruitment strategy and informed consent process

- Recruitment to the cohort will take place at Fiocruz ambulatory, FMT-HVD and MHC where a study nurse will be based. Informed consent will be explained by trained research nurses at the time of recruitment. All adult men or women, irrespectively of knowing their ZIKV diagnosis status, corresponding to inclusion criteria should hence be informed about the study and invited to participate, providing informed consent in writing.
- Household/sexual contacts of individuals diagnosed with ZIKV infection (index case) and who accepted to take part in the study, will also be invited to participate. Invitation to participate in the study will be extended to symptomatic or asymptomatic individuals aged 18 years and above. They will either come to the clinic or receive the visit of a trained research staff who will explain the study, assess eligibility and seek informed consent or will be invited to attend the collaborating clinics where the index case diagnosis was performed.
- Participants will also be informed that they are free to choose whether or not to participate and their decision will not affect their medical care. They will also be informed they can withdraw from the study at any time of their choice if they wish to do so.
- Medical management will not be compromised by the study procedures and priority will be given to tests required for medical management.

##### Reimbursement or compensation to study participants

Participants will receive reimbursement to cover the cost of transport and a meal. This will happen at every visit to the study clinics. As incentive payments are illegal under Brazilian law an offer of reimbursement will be made through provision of meal and transport tickets as needed.

##### Applicability of results

The results of this study will provide evidence base for recommendation on prevention of ZIKV transmission. This will be applicable to both the current and future outbreaks that may occur.

##### Plans for dissemination and use of project results

Throughout the research study, results will be disseminated principally via principal investigators and respective teams. Meetings can be organized with study staff and participants as well as in community as necessary. Community meeting can be conducted by the study team.

## Discussion

Both men and women are significantly affected by the ZIKV epidemic. Women are potentially more vulnerable to the risk of sexual transmission of ZIKV, given both their biological vulnerability related to the vaginal mucosa and structural gender inequities that might place women in an inferior position when it comes to negotiating condom use.

In addition, due to the potential devastating complications associated with infection by the ZIKV, women and in particular pregnant women, are put under pressure and emotionally affected not only by the possibility of an unwanted pregnancy but also its social and economic consequences as they will bear the impact of caring for these children. In addition, Brazil has a restrictive legislation on abortion putting women at higher risk of death in consequence of clandestine abortion.

The findings about the persistence and duration of the virus in different body fluids will assist with the recommendation on the period during which men should use condom to prevent transmission to their sexual partners and that females should consider deferring pregnancy. Breastfeeding will be addressed according to the evidence base at the time of the counselling. At the moment recommendations on breastfeeding have not changed. Infants should start breastfeeding within 1 hour of birth and continue for 6 months exclusively and up to 2 years of age or beyond, after the introduction of complementary food.

Participants will be offered timely risk reduction counselling by trained nurses on prevention of transmission or acquisition of ZIKV. In addition, all participants in need of specific health services will be referred to the appropriate clinic/hospital for care and management. HIV positive participants will be referred to the national program that includes HIV counselling and treatment services.

Women will be offered pregnancy testing (pregnant women will not be included in the study). The study nurses will be trained and made aware of the significant mental anguish about reproductive issues that women experience during the ZIKV outbreak.

Mothers whose breast milk is found to be positive for ZIKV will be counselled according to the most up-to-date information about ZIKV infection and guidelines.

The success of the project will depend on the willingness of eligible patients to take part in the study by providing all body fluid specimens for a period of 12 months. Cases will be recruited according to the demand until the necessary sample size is reached. The type of specimens to be collected and tested will comprise semen, vaginal secretion and menstrual blood. In addition, participants will be asked intimate questions that might make them feel uncomfortable and reluctant to partake.

In order to overcome this potential drawback, only well trained professionals who are comfortable to discussing sexual behavior will be interviewing participants. In addition participants will be reassured that all information provided will be kept confidential.

This prolonged longitudinal follow-up of ZIKV infected persons with regular biologic testing and data collection will offer a unique opportunity to investigate the presence and persistence of ZIKV in various biologic compartments, their clinical and immunological correlates as well as the possibility of ZIKV reactivation/reinfection over time. This valuable information will substantially contribute to the body of knowledge on ZIKV infection and will serve as a base for the development of more effective recommendation on the prevention of ZIKV transmission.

## Additional files

Additional file 1: Questionnaire_screening. (PDF 370 kb)

Additional file 2: Questionnaire_enrolment_female. (PDF 460 kb)

Additional file 3: Questionnaire_enrolment_male. (PDF 444 kb)

Additional file 4: Questionnaire_follow.up_female. (PDF 311 kb)

Additional file 5: Questionnaire_follow.up_male. (PDF 299 kb)

Additional file 6: Consultation_form_1st_visit. (PDF 608 kb)

Additional file 7: Consultation_form_2st_visit. (PDF 350 kb)

## Abbreviations

Fiocruz: Fundação Oswaldo Cruz
HBV: Hepatitis B virus
HCV: Hepatitis C virus
HIV: Human Immunodeficiency virus
IgG: Immunoglobulin G
IgM: Immunoglobulin M
MHC: Municipal Health Center
PAHO: Pan American Health Organization
PBMC: Peripheral Blood Mononuclear Cell
RNA: Ribonucleic acid
RT-PCR: Reverse transcriptase-polymerase chain reaction
SST: Serum Separator Tube
UPA: Unidade de Pronto Atendimento (Walk-in Centers)
USA: United States of America
WHO: World Health Organization
ZIKV: Zika virus
