# Supplementary material for: Study on the persistence of Zika virus (ZIKV) in body fluids of patients with ZIKV infection in Brazil
Source: BMC Infect Dis. 2018 Jan 22;18:49. doi: 10.1186/s12879-018-2965-4 (PMC5778641; doi:10.1186/s12879-018-2965-4)
Supplement: Supplementary file 1 — Questionnaire_screening. (PDF 370 kb) [file 12879_2018_2965_MOESM1_ESM.pdf]

Site ID: ☐☐
(1 = Rio, 2 = Manaus, 3 = Recife)

Unique participant ID: ☐☐☐☐

Participant type: ☐
(1 = Index, 2 = Contact symptomatic, 3 = Contact asymptomatic)

GAL number: ☐☐☐☐☐☐☐☐☐☐☐

If Contact, Screening number of Index ☐☐☐

SCREENING INFORMATION

1. a) Date of screening:

**Day**		**Month**			**Year**		

b) Screened by (nurse name): Initials ☐☐☐

1 = No 2 = Yes

2. a) Consent for enrolment: ☐

b) Consent for follow up of household members: ☐

ZIKA SYMPTOMS

1 = No 2 = Yes

3. Zika symptoms

a) Temperature °C ☐☐ ☐

b) Rash ☐

c) Mialgia ☐

d) Arthralgia ☐

e) Conjunctivitis ☐

f) Pruritus ☐

g) Peri-auricular edema ☐

Thank you for participating in the study. I will be conducting your interview today and it will last around 10 minutes. I ask all participants in this study the same questions. All of your answers are confidential. I will mark a response to every question. It is ok to say "I prefer not to answer" at any time and we will move on to the next question. You can also ask me to go back, or repeat any questions. I would like to remind you that your participation in the study is completely confidential. Are you comfortable to proceed with the interview now?

I would like to start by asking you a few questions about yourself and your family.

SOCIAL DEMOGRAPHIC DATA

4. Participant's gender *(Do you define yourself as):* ☐

1 = Male 3 = Transgender
2 = Female 4 = Transvestite

5. What is your age today? **Years** ☐☐
(Mark ***99*** if ***refused***)

6. What is your date of birth?
(Mark ***8888*** in years if unknown and ***9999*** if question refused)

**Day**		**Month**			**Year**		

Site ID: ☐☐ Unique participant ID: ☐☐☐☐

7. What is your race/skin colour? (Read and choose only one option) ☐

1 = White
2 = Black
3 = Parda
4 = Indigenous
5 = Other
8 = Don't know
9 = Refused

If ***Other*** , specify: ____________________

8. What is your occupation? ____________________

9. What is your level of education? ☐

1 = No school (*Skip to Q10)*
2 = Primary - complete
3 = Primary - incomplete
4 = Secondary complete
5 = Secondary incomplete
6 = University
7 = Post graduated
8 = Don’t know
9 = Refused (*Skip to Q10)*

10. Years of concluded study: Years ☐☐

11. What is your current relationship status? ☐

1 = Single
2 = Married
3 = Widowed
4 = Long-term relationship
5 = Separated or divorced
6 = Other
8 = Don’t know
9 = Refused

If ***Other*** , specify:

12. Remarks

____

____

____

	**Day**		**Month**			**Year**			
Date SCR completed:									

Signature: ____________________
