# Supplementary material for: Study on the persistence of Zika virus (ZIKV) in body fluids of patients with ZIKV infection in Brazil
Source: BMC Infect Dis. 2018 Jan 22;18:49. doi: 10.1186/s12879-018-2965-4 (PMC5778641; doi:10.1186/s12879-018-2965-4)
Supplement: Supplementary file 2 — Questionnaire_enrolment_female. (PDF 460 kb) [file 12879_2018_2965_MOESM2_ESM.pdf]

	Persistence of Zika virus in body fluids of patients with Zika virus infection<br><br>Enrollment questionnaire - Female	WER<br>Page 1/9<br>V1.2 (08Sept2016)

Site ID: ☐
(1 = Rio, 2 = Manaus, 3 = Recife)

Unique participant ID: ☐☐☐☐

Participant type: ☐
(1 = Index, 2 = Contact symptomatic, 3 = Contact asymptomatic)

GAL number: ☐☐☐☐☐☐☐☐☐☐☐

If Contact, number of Index ☐☐☐

RECRUITMENT INFORMATION

1. a) Date of screening:

Day		Month		Year			

b) Screened by (nurse name): Initials ☐☐☐

1 = No 2 = Yes

2. a) Consent for enrolment: ☐

b) Consent for follow up of household members: ☐

ZIKA SYMPTOMS

1 = No 2 = Yes

3. Zika symptoms

a) Temperature °C ☐☐ ☐

b) Rash ☐

c) Mialgia ☐

d) Arthralgia ☐

e) Conjunctivitis ☐

f) Pruritus ☐

g) Peri-auricular edema ☐

Thank you for participating in the study. I will be conducting your interview today and it will last around 60 minutes. I ask all participants in this study the same questions. All of your answers are confidential. I will mark a response to every question. It is ok to say "I prefer not to answer" at any time and we will move on to the next question. You can also ask me to go back, or repeat any questions. I would like to remind you that your participation in the study is completely confidential.
Are you comfortable to proceed with the interview now?

I would like to ask you some questions about your home and some items within your home.
When answering the questions, please, consider only items which are working, including those kept but not used. If they are not working, consider only those that you have intention to fix over the next 6 months.

4. Are you living alone or with other people? ☐
   1 = Alone
   2 = Other people

5. Can you, please, choose the option that best describes your household? ☐
   1 = Live alone
   2 = Couple with children under 16 years
   3 = Couple with children over 16 years
   4 = No partner with children under 16 years
   5 = No partner with children over 16 years
   6 = No partner with children under and over 16 years
   7 = No partner, no children, with relatives
   8 = No partner, no children, with aggregates
   9 = Couple without children
   10 = Couple without children with relatives

Site ID: ☐

(1 = Rio, 2 = Manaus, 3 = Recife)

Unique participant ID: ☐☐☐☐

6. How many people live in your house, including yourself? ☐☐

7. Are you the head of the family? ☐

1 = No
9 = Refused
2 = Yes

8. What is your main source of income? ☐

1 = Employed with monthly salary
2 = Temporary employed with salary
3 = Self employed
4 = On benefit due to illness
5 = Retired due to illness
6 = Retired for time of work
7 = Unemployed
8 = Another source
9 = Refused

9. Your residence is: ☐

1 = Owned by you
2 = Rented
3 = Lended

10. Considering the part of the street where you live you would say that it is: ☐

1 = Paved/asphalted
2 = Gravel
9 = Refused

11. What is your main source of water? ☐

1 = Internal pipes
2 = No pipes
3 = No water service
4 = Well
4 = Piped water outside your home
5 = Other source
8 = Don't know
9 = Refused

If ***Other*** , specify: ______________________________

12. How is the waste collected from your home? ☐

1 = Directly
1 = No service
8 = Don't know
9 = Refused

Please report how many of the following apply to you situation , using the codes here under

0 = 0   1 = 1   2 = 2   3 = 3   4 = 4   5 = 5 or more   9 = Refused

13. Bathrooms in your home? ☐

14. Servents working in your home? ☐

15. Cars you own? (This does not include taxi or cars for commercial use) ☐

16. Motorbikes you own? (This does not include motorbike for professional use) ☐

17. Computers you have in your home? ☐

18. Dish washers you have in your home? ☐

Site ID: ☐ Unique participant ID: ☐☐☐☐

19. Washing machines you have in your home? ☐

20. Tumble dryers you have in your home? ☐

21. Fridges you have in your home? ☐

22. Freezers you have in your home? (Standalone or as part of the fridge) ☐

23. DVD players you have in your home? (This does not include your car's DVD) ☐

24. Microwaves oven you have in your home? ☐

EPIDEMIOLOGICAL DATA

Next I would like to ask you about history of travel and your current symptoms.

25. a) Have you travelled in the previous 30 days? ☐
1 = No *(Skip to Q21)* 2 = Yes 9 = Refused

b) How many times have you travelled in the previous 30 days? ☐
1 = 1 2 = 2 3 = 3 4 = 4 5 = 5 or more 9 = Refused

c) Date you started your last travel:

Day	Month	Year

d) Date you returned from your last travel:

Day	Month	Year

e) Destination of your last travel: ______________________________

f) Date of previous travels in the last 30 days (more recent travel first)

1

Day	Month	Year

2

Day	Month	Year

3

Day	Month	Year

g) Date you returned from your previous travels in the last 30 days (more recent travel first)

1

Day	Month	Year

2

Day	Month	Year

3

Day	Month	Year

h) Destination of your previous travel in the last 30 days (more recent first):

1 ______________________________

2 ______________________________

3

Site ID: ☐ Unique participant ID: ☐☐☐☐

26. a) Do you know anyone else presenting two or more of the following symptoms? ☐
(Rash, fever, joint pain, muscle pain, pruritus, conjunctivitis -red eyes, headache)

1 = No 8 = Don't know
2 = Yes 9 = Refused

If ***No***, ***Don't know*** or ***Refused***, go to ***Q28***

b) If ***Yes***, where are they from, who are they and how many?

	Where are they from?<br>**1 = No 2 = Yes**	If ***Yes***, How many are they?
1. Home	☐	☐☐
2. Partner	☐	☐☐
3. Neighbour	☐	☐☐
4. Work	☐	☐☐

27. Could you, please, tell me who are these persons and the timing of their symptoms in relation to your?
(Nurses should capture information on sexual partners, siblings, children).
If participant refuses to specify, write "Refused" under relationship

**Relationship**<br>1 = Yes 2 = No	**Did this person have Zika before of after you?**<br>1 = Before case 2 = After case 9 = Refused
a) Husband	☐
b) Wife	☐
c) Sexual partner	☐
d) Father	☐
e) Mother	☐
f) Brother	☐
g) Sister	☐
h) Grandparent	☐
i) Other	☐

If ***Other***, specify: ______________________________

Site ID: ☐ Unique participant ID: ☐☐☐☐

Before moving to the next question make sure the number reported in question 20b is consistent with the number of rows completed in question 21.

I would like to ask you some personal questions. Please be reassured you that all your answers will remain confidential. If you feel uncomfortable to answer any question, it is OK to say "I prefer not to answer" at any time and we will move on to the next question. We ask every participant in the study the same questions. By sharing this private information about your personal life, you will help us to better understand whether Zika can be passed on through sex and when it is safe to have sex after having Zika disease.
By sex I mean vaginal, anal, oral sex with another person.

28. a) Have you had sexual relationship (vaginal, anal, oral) during the last 12 months? ☐

1 = No 8 = Don't know
2 = Yes 9 = Refused

b) Did your partner use condom during your last sexual relationship? ☐

1 = No 2 = Yes

29. a) Are you currently using any contracpetive method other than condom? ☐

1 = No (***Skip*** to ***Q25***) 2 = Yes

b) What method other than condom are you using? (You may choose up to 3 answers) ☐☐

01 = Pill/oral contraception 06 = Spermicide
02 = Intrauterine device 07 = Following day pill
03 = Injection/hormone shot 08 = Refused
04 = Implant 09 = Interrupted coitus
05 = Female/male condom 10 = Other

If ***Other*** , specify: ______________________________

30. a) Are you and your sexual partner planning to get pregnant? ☐

1 = No 8 = Don't know
2 = Yes 9 = Refused

b) Have you received information about the health consequences of Zika virus infection during pregnancy? ☐

1 = No 8 = Don't know
2 = Yes 9 = Refused

31. Would you consider to abstain from sex while Zika outbreak is ongoing? ☐

1 = No 8 = Don't know
2 = Yes 9 = Refused

32. Did you receive advice on contraception since Zika outbreak started? ☐

1 = No 8 = Don't know
2 = Yes 9 = Refused

Site ID: ☐ Unique participant ID: ☐☐☐☐

33. a) Before today, did you receive information from anyone about when it is safe for someone who had Zika infection to have sex? ☐

1 = No (Skip to Q29) 8 = Don't know

2 = Yes 9 = Refused

b) If ***Yes*** , from whom? ☐

1 = Friends or family members 3 = Public health or government officials

2 = Leaders in the community 4 = Other

If ***Other*** , specify: ____________________

c) If ***Yes*** , what advice did you receive?

1 = No 2 = Yes

1.a) = Someone who had Zika should abstain from having sex ☐

b) If ***Yes*** , ask for how long Months ☐☐

2. a) Sexual partner to use condom: ☐

b) If ***Yes*** , ask for how long (months): ☐☐

3. It is safe to have sex intercourse immediately after Zika infection: ☐

4. Any other advice: ☐

If ***Other*** , specify: ____________________

COMORBIDITIES

Now I would like to ask you about health problems that you may have had in the past.

34. When was the last time that you saw a doctor? ☐

1 = In the last 12 months 4 = I never saw a doctor

2 = Between 1 and 2 years 8 = Don’t know

3 = More than 2 years 9 = Refused

35. In general how would you classify your health status? ☐

1 = Very good 5 = Very bad

2 = Good 8 = Don’t know

3 = Regular 9 = Refused

4 = Bad

Site ID: ☐ Unique participant ID: ☐☐☐☐

36. Do you suffer from any of the following conditions below?
1 = No 2 = Yes

a) Anemia: ☐
b) Diabetes: ☐
c) Rinitis: ☐
d) CPOD (chronic obstructive pulmonary disease): ☐
e) CVD (cardio vascular disease): ☐
f) Hypertension: ☐
g) Past or sequelae of stroke: ☐
h) Arthritis: ☐
i) Joint and muscle diseases: ☐
j) Chronic hepatitis (B or C): ☐
k) Kidney disease: ☐
l) HIV: ☐
m) Malaria ☐
n) Falciform anaemia: ☐
o) Other: ☐
If ***Other*** , specify: ____________

37. Are you taking any medication regularly? ☐
1 = No 8 = Don't know
2 = Yes 9 = Refused

If ***Yes,*** specify: ____________

38. a) Do you have any allergy? ☐
1 = No 8 = Don't know
2 = Yes 9 = Refused

b) If ***Yes*** , please give details: ____________

39. a) Are you pregnant? ☐
1 = No 8 = Don't know
2 = Yes 9 = Refused

b) If ***Yes*** , date last period:

Day		Month		Year			

c) If ***Yes*** , probable date of delivery:

Day		Month		Year			

40. a) Have you ever been sick with Zika before? ☐
1 = No 8 = Don't know
2 = Yes 9 = Refused

b) If ***Yes*** , how many times have you been sick with this Zika before?

Day		Month		Year			

____

Site ID: ☐ Unique participant ID: ☐☐☐☐

41. a) Have you ever been sick with Dengue before? ☐
1 = No 8 = Don't know
2 = Yes 9 = Refused

b) If ***Yes*** , how many times have you been sick with this Dengue before? ____________

Day		Month		Year			

Day		Month		Year			

Day		Month		Year			

42. a) Have you ever been sick with Chikungunya before? ☐
1 = No 8 = Don't know
2 = Yes 9 = Refused

b) If ***Yes*** , how many times have you been sick with this Chikungunya before?

Day		Month		Year			

43. a) Have you ever been sick with West Nile virus before? ☐
1 = No 8 = Don't know
2 = Yes 9 = Refused

b) If ***Yes*** , how many times have you been sick with West Nile virus before?

Day		Month		Year			

44. a) Have you ever been sick with Oropouche virus before? ☐
1 = No 8 = Don't know
2 = Yes 9 = Refused

b) If ***Yes*** , how many times have you been sick with Oropouche virus before?

Day		Month		Year			

45. a) Have you ever been sick with Mayaro virus before? ☐
1 = No 8 = Don't know
2 = Yes 9 = Refused

b) If ***Yes*** , how many times have you been sick with Mayaro virus before?

Day		Month		Year			

VACCINES

I would like to ask you about the vaccines that you have received. Could you, please, tell me if you ever have taken one of the vaccines below? If yes and you don't remember the exact date, please, give the approximate date.

46. Have you received a vaccine against Yellow Fever in the last 10 years? ☐
1 = No 8 = Don't know
2 = Yes 9 = Refused

b) If ***Yes*** , date:

Day		Month		Year			

	Persistence of Zika virus in body fluids of patients with Zika virus infection<br><br>Enrollment questionnaire - Female	WER<br>Page 9/9<br>V1.2 (08Sept2016)

Site ID: ☐ Unique participant ID: ☐☐☐

47. Have you received any vaccine(s) against **Rubela** since you are an adult? ☐

1 = No 8 = Don't know
2 = Yes 9 = Refused

b) If ***Yes*** , date:

Day		Month		Year			

Day		Month		Year			

Day		Month		Year			

48. Have you received any vaccine(s) against **diphtheria and tetanus (DT)** since you are an adult, including during pregnancy? ☐

1 = No 8 = Don't know
2 = Yes 9 = Refused

b) If ***Yes*** , date:

Day		Month		Year			

Day		Month		Year			

Day		Month		Year			

49. Have you received any vaccine(s) against **Hepatitis B** since you are an adult, including during pregnancy? ☐

1 = No 8 = Don't know
2 = Yes 9 = Refused

b) If ***Yes*** , date:

Day		Month		Year			

Day		Month		Year			

Day		Month		Year			

50. Have you received any vaccine(s) against **Influenza** since you are an adult, including during pregnancy? ☐

1 = No 8 = Don't know
2 = Yes 9 = Refused

b) If ***Yes*** , date:

Day		Month		Year			

Day		Month		Year			

Day		Month		Year			

Date WER completed:

Day		Month		Year			

Signature: ____________________
