# Supplementary material for: Study on the persistence of Zika virus (ZIKV) in body fluids of patients with ZIKV infection in Brazil
Source: BMC Infect Dis. 2018 Jan 22;18:49. doi: 10.1186/s12879-018-2965-4 (PMC5778641; doi:10.1186/s12879-018-2965-4)
Supplement: Supplementary file 4 — Questionnaire_follow.up_female. (PDF 311 kb) [file 12879_2018_2965_MOESM4_ESM.pdf]

Site ID: ☐☐ Unique participant ID: ☐☐☐☐

(1 = Rio, 2 = Manaus, 3 = Recife)

Participant type: ☐ GAL number: ☐☐☐☐☐☐☐☐☐☐☐

(1 = Index, 2 = Contact symptomatic, 3 = Contact asymptomatic)

If Contact, Screening number of Index ☐☐☐

ZIKA INFECTION CONVALESCENCE

Thank you for participating in the study. I will be conducting your interview today and it will last around 10 minutes. I ask all participants in this study the same questions. All of your answers are confidential. It is ok to say "I prefer not to answer"at any time and we will move on to the next question. You can also ask me to go back or repeat any question. I would like to remind you that your participation is completely confidential.
Are you comfortable proceeding with the interview now?

I would like to talk to you about your health and wellbeing today after your recovery from Zika infection.
(If asymptomatic case, skip to question 3)

1. Compared to your last visit how is your overall health and well being? ☐
   1 = The same as how I felt at my last study visit
   2 = Worst than how I felt at my last study visit
   3 = Better than how I felt at my last study visit
   4 = Not applicable
   8 = Don't know
   9 = Refused

2. a) After recovering from Zika do you have any new health problems? ☐
   1 = No
   2 = Yes
   3 = Not applicable
   8 = Don't know
   9 = Refused

   b) If ***Yes*** , please, specify the problems you are experiencing:
   1 = No
   2 = Yes

   1. General symptoms (fever, weight loss, loss of appetite, feeling tired) ☐
   2. Eye problems (itching, ocular redness, eye lid inflammation, blurred vision, loss of vision) ☐
   3. Joint problems ☐
   4. Abdominal pain ☐
   5. Headache ☐
   6. Neurological problems (loss of strength in the arms or legs, loss of balance) ☐
   7. Skin problems ☐
   8. Psychiatric/psychological problems (delusions, hallucinations, depression, anxiety) ☐
   9. Other ☐

   If ***Other*** specify***:***

Site ID: ☐☐ Unique participant ID: ☐☐☐☐

3. a) After your last study visit have you been hospitalized? ☐

1 = No 8 = Don't know

2 = Yes 9 = Refused

If ***No*** , ***Don't know*** or ***Refused*** , Go to ***Q4a***

b) What symptoms did you have? ______________________

c) What day did you go to the hospital?

Day		Month			Year			

d) How many days did you stay in the hospital? **Days** ☐☐

e) What was the diagnosis? ______________________

f) What treatment did you receive? ______________________

4. a) After your last study visit has anyone in your home/sexual partner got Zika virus infection? ☐

1 = No 8 = Don't know

2 = Yes 9 = Refused

b) If **Yes**, can you tell me who are these persons?

Relationship

1 = No 2 = Yes

a) Husband ☐

b) Wife ☐

c) Sexı Sexual partner ☐

d) Father ☐

e) Mother ☐

f) Brother ☐

g) Sister ☐

h) Grandparent ☐

i) Other ☐

If ***Other*** , specify: ______________________

5. Are you using any contraception method? May choose up to 3 answers ☐☐ ☐☐ ☐☐

0 = None

1 = Pill/oral contraception

2 = Intrauterine device

3 = Injection/hormone shot

4 = Implant

5= Female/male condom

6 = Spermicide

7 = Following day pill

8 = Interrupted coitus

9 = Other

99 = Refused

If **Other,** specify: ______________________

Site ID: ☐☐ Unique participant ID: ☐☐☐☐

6. a) After you last study visit did you have sex? ☐

1 = No 8 = Don't know

2 = Yes 9 = Refused

b) If ***Yes*** , did your partner use condom during your last sex relationship? ☐

c) How many times did you have sex? ☐

1 = Every day 3 = 3 or more times a week

2 = 2 times a week 4 = Once per week

7. a) After your last visit have you had your menstrual period?

1 = No 8 = Don't know

2 = Yes 9 = Refused

3 = Menopause

If ***No*** , ***Don't know, Menopause*** or ***Refused*** , Go to ***Q9a***

b) If ***Yes*** , date last period:

Day		Month			Year			

c) If ***Yes*** , have your period changed? ☐

1 = Yes, my period became different 8 = Don't know

2 = No, I have not notice any difference in my period 9 = Refused

3 = I am pregnant

d) If ***Yes*** , how?

1 = No 2 = Yes

1. It is more difficult to predict the first day of bleeding/period ☐

2. Shorter periods ☐

3. Longer periods ☐

4. Spotting or repeat bleeding in the same month ☐

5. Less frequent periods ☐

6. Other ☐

If ***Other*** , specify: ____________________

8. a) After your last study visit did you become pregnant? ☐

1 = No 8 = Don't know

2 = Yes 9 = Refused

Site ID: ☐☐ Unique participant ID: ☐☐☐☐

8. CONTINUED

b) If ***Yes***, how did you find out?

1 = No 8 = Don't know
2 = Yes 9 = Refused

1. By doing a test on urine? ☐
2. By doing a test on blood? ☐
3. By doing an ultrasound ☐

c) If ***Yes***, how many months pregnant are you? **Months** ☐

9. Currently do you have any of the following symptoms?

1 = No 8 = Don't know
2 = Yes 9 = Refused

a) Lower abdominal pain ☐

b) Vaginal discharge ☐

c) Ulcers in genital or anal region ☐

d) Discharge or pain in the rectum ☐

e) Genital itching ☐

f) Growth in genital or anal region ☐

g) Other ☐

If ***Other***, specify: ____________________

Thank you very much for participating in the survey. Do you have any questions, comments or concerns about what we discussed today?

Remarks: ____________________

____

____

____

Day		Month		Year			

Date WFU completed: Signature: ____________________
