# Supplementary material for: Study on the persistence of Zika virus (ZIKV) in body fluids of patients with ZIKV infection in Brazil
Source: BMC Infect Dis. 2018 Jan 22;18:49. doi: 10.1186/s12879-018-2965-4 (PMC5778641; doi:10.1186/s12879-018-2965-4)
Supplement: Supplementary file 5 — Questionnaire_follow.up_male. (PDF 299 kb) [file 12879_2018_2965_MOESM5_ESM.pdf]

Site ID: ☐☐ Unique participant ID: ☐☐☐☐

(1 = Rio, 2 = Manaus, 3 = Recife)

Participant type: ☐ GAL number: ☐☐☐☐☐☐☐☐☐☐☐

(1 = Index, 2 = Contact symptomatic, 3 = Contact asymptomatic)

If Contact, number of Index ☐☐☐

ZIKA INFECTION CONVALESCENCE

Thank you for participating in the study. I will be conducting your interview today and it will last around 10 minutes. I ask all participants in this study the same questions. All of your answers are confidential. It is ok to say "I prefer not to answer"at any time and we will move on to the next question. You can also ask me to go back or repeat any question. I would like to remind you that your participation is completely confidential.
Are you comfortable proceeding with the interview now?

I would like to talk to you about your health and wellbeing today after your recovery from Zika infection.
(If asymptomatic case, skip to question 3)

1. Compared to your last visit how is your overall health and well being? ☐
   - 1 = The same as how I felt at my last study visit
   - 2 = Worst than how I felt at my last study visit
   - 3 = Better than how I felt at my last study visit
   - 8 = Don't know
   - 9 = Refused

2. a) After recovering from Zika do you have any new health problems? ☐
   - 1 = No
   - 2 = Yes
   - 8 = Don’t know
   - 9 = Refused

   b) If ***Yes*** , please, specify the problems you are experiencing:
   1 = No 2 = Yes

   1. General symptoms (fever, weight loss, loss of appetite, feeling tired) ☐
   2. Eye problems (itching, ocular redness, eye lid inflammation, blurred vision, loss of vision) ☐
   3. Joint problems ☐
   4. Abdominal pain ☐
   5. Headache ☐
   6. Neurological problems (loss of strength in the arms or legs, loss of balance) ☐
   7. Skin problems ☐
   8. Psychiatric/psychological problems (delusions, hallucinaties, depression, anxiety) ☐
   9. Other ☐

   If ***Other,*** specify***:*** ______________________________

Site ID: ☐☐

Unique participant ID: ☐☐☐☐

3. a) After your last study visit have you been hospitalized? ☐

   1 = No   8 = Don't know
   2 = Yes   9 = Refused

   If ***No*** , ***Don't know*** or ***Refused*** , Go to ***Q4a***

   b) What symptoms did you have? ______________________

   c) What day did you go to the hospital?

Day		Month		Year			

   d) How many days did you stay in the hospital? Days ☐☐

   e) What was the diagnosis? ______________________

   f) What treatment did you receive? ______________________

4. a) After your last study visit has anyone in your home/sexual partner got Zika virus infection? ☐

   1 = No   8 = Don't know
   2 = Yes   9 = Refused

   b) If **Yes**, can you tell me who are these persons?

   Relationship

   1 = No   2 = Yes

   a) Husband ☐   f) Brother ☐
   b) Wife ☐   g) Sister ☐
   c) Sexı Sexual partner ☐   h) Grandparent ☐
   d) Father ☐   i) Other ☐
   e) Mother ☐   If ***Other*** , specify: ______________________

5. After recovering from Zika, do you have the same level of sexual desire as before you were sick? ☐

   (If asymptomatic case, skip to question 6)

   1 = No   8 = Don't know
   2 = Yes   9 = Refused

6. a) After you last study visit did you have sex? ☐

   1 = No   8 = Don't know
   2 = Yes   9 = Refused

   b) If ***Yes*** , did you use condom during your last sex relationship? ☐

   c) How many times did you have sex? ☐

   1 = Every day   5 = Once per month
   2 = 2 times a week   6 = Once
   3 = 3 or more times a week   8 = Don't know
   4 = Once per week   9 = Refused

Site ID: ☐☐ Unique participant ID: ☐☐☐☐

Participant type: ☐ GAL number: ☐☐☐☐☐☐☐☐☐☐☐

(1 = Index, 2 = Contact symptomatic, 3 = Contact asymptomatic)

7. Currently do you have any of the following symptoms?

1 = No 8 = Don't know

2 = Yes 9 = Refused

a) Penile/urethral discharge ☐

b) Ulcers in genital or anal region ☐

c) Lower abdominal pain ☐

d) Groin swelling ☐

e) Scrotal swelling ☐

f) Discharge or pain in the rectum ☐

g) Genital itching ☐

h) Growth in genital or anal region ☐

i) Blood in your sperm ☐

j) Other ☐

If ***Other***, specify: ______________________

Thank you very much for participating in the survey. Do you have any questions, comments or concerns about what we discussed today?

Remarks: ______________________

____

____

Date MFU completed:	Day		Month		Year				Signature: ______________________
