# Supplementary material for: Study on the persistence of Zika virus (ZIKV) in body fluids of patients with ZIKV infection in Brazil
Source: BMC Infect Dis. 2018 Jan 22;18:49. doi: 10.1186/s12879-018-2965-4 (PMC5778641; doi:10.1186/s12879-018-2965-4)
Supplement: Supplementary file 6 — Consultation_form_1st_visit. (PDF 608 kb) [file 12879_2018_2965_MOESM6_ESM.pdf]

Symptoms/Signs version 1.1 12/set/16	Persistence of Zika virus in body fluids of patients with Zika virus infection<br>Consultation form - 1st consult	CON1<br>Page 1/8<br>v1.1(12/09/2016

Site ID: ☐ Unique participant ID: ☐☐☐☐

(1 = Rio, 2 = Manaus, 3 = Recife)

Participant type: ☐ GAL number: ☐☐☐☐☐☐☐☐☐☐☐

(1 = Index, 2 = Contact symptomatic, 3 = Contact asymptomatic)

If household contact/sexual partner, number of Index: ☐☐☐☐

VITAL SIGNS

1. Date of first appointment

Day		Month			Year			

2. Main complaint

____

3. Date of first symptoms

Day		Month			Year			

4. a) Does the patient has fever? ☐

1 = No (***Skip to Q5***) 2 = Yes

b) Fever onset ☐

1 = sudden 2 = gradual

c) Highest temperature measured so far °C ☐☐, ☐

d) Current temperature °C ☐☐, ☐

e) Date of the end of fever

Day		Month			Year			

5. Heart rate per minute ☐☐☐

6. Respiratory rate per minute ☐☐

7. a) Blood pressure sitted (Systolic/Dyastolic) mmHg ☐☐☐ / ☐☐☐

b) Bood pressute lying down (Systolic/Dyastolic) mmHg ☐☐☐ / ☐☐☐

8. Weight Kg ☐☐☐, ☐

9. Height m ☐, ☐☐

Site ID: ☐ Unique participant ID: ☐☐☐☐

SYMPTOMS

1 = No 2 = Yes

10. a) Chill ☐

b) Date of onset

Day	Month	Year

Day	Month	Year

11. a) Headache ☐

b) Date of onset

Day	Month	Year

Day	Month	Year

12. a) Photophobia ☐

b) Date of onset

Day	Month	Year

Day	Month	Year

13. a) Retro orbital pain ☐

b) Date of onset

Day	Month	Year

Day	Month	Year

14. a) Ocular burning sensation ☐

b) Date of onset

Day	Month	Year

Day	Month	Year

15. a) Oropheryngeal pain ☐

b) Date of onset

Day	Month	Year

Day	Month	Year

16. a) Hoarseness ☐

b) Date of onset

Day	Month	Year

Day	Month	Year

17. a) Change in taste ☐

b) Date of onset

Day	Month	Year	Day	Month	Year

Symptoms/Signs version 1.1 12/set/16	Persistence of Zika virus in body fluids of patients with Zika virus infection Consultation form - 1st consult	CON1 Page 3/8 v1.1(12/09/2016

Site ID: ☐ Unique participant ID: ☐☐☐☐

18. a) Earache ☐

b) Date of onset

Day	Month	Year	Day	Month	Year

19. a) Coryza ☐

b) Date of onset

Day	Month	Year	Day	Month	Year

20. a) Nasal congestion ☐

b) Date of onset

Day	Month	Year	Day	Month	Year

21. a) Sudoresis ☐

b) Date of onset

Day	Month	Year	Day	Month	Year

22. a) Prostration ☐

b) Date of onset

Day	Month	Year	Day	Month	Year

23. a) Myalgia ☐

b) Date of onset

Day	Month	Year	Day	Month	Year

24. a) Lombalgia ☐

b) Date of onset

Day	Month	Year	Day	Month	Year

25. a) Muscular weakness ☐

b) Date of onset

Day	Month	Year

Day	Month	Year

Site ID: ☐

Unique participant ID: ☐☐☐☐

26. a) Arthralgia ☐

b) Date of onset

Day	Month	Year

Day	Month	Year

d) Site of arthalgia

1. Shoulder ☐
4. hip ☐
2. Elbow ☐
5. Ankle ☐
3. Knee ☐

27. a) Anorexia ☐

b) Date of onset

Day	Month	Year

Day	Month	Year

28. a) Nausea ☐

b) Date of onset

Day	Month	Year

Day	Month	Year

29. a) Vomit ☐

b) Date of onset

Day	Month	Year

Day	Month	Year

30. a) Abdominal pain ☐

b) Date of onset

Day	Month	Year

Day	Month	Year

31. a) Choluria ☐

b) Date of onset

Day	Month	Year

Day	Month	Year

32. a) Dysuria ☐

b) Date of onset

Day	Month	Year

Day	Month	Year

Site ID: ☐ Unique participant ID: ☐☐☐☐

33. a) Dyarrhoea ☐

b) Date of onset

Day	Month	Year

Day	Month	Year

34. a) Dyspnoea ☐

b) Date of onset

Day	Month	Year

Day	Month	Year

35. a) Cough ☐

b) Date of onset

Day	Month	Year

Day	Month	Year

36. a) Haemarrhage ☐

b) Date of onset

Day	Month	Year

Day	Month	Year

37. a) Epistaxe ☐

b) Date of onset

Day	Month	Year

Day	Month	Year

38. a) Gum bleeding ☐

b) Date of onset

Day	Month	Year

Day	Month	Year

39. a) Metrorrhagia ☐

b) Date of onset

Day	Month	Year

Day	Month	Year

40. a) Hematuria ☐

b) Date of onset

Day	Month	Year		Day	Month	Year

Site ID: ☐ Unique participant ID: ☐☐☐☐

41. a) Hematemesis ☐

b) Date of onset

Day	Month	Year		Day	Month	Year

42. a) Melena ☐

b) Date of onset

Day	Month	Year		Day	Month	Year

43. a) Hemoptysis ☐

b) Date of onset

Day	Month	Year		Day	Month	Year

44. a) Erythema ☐

b) Date of onset

Day	Month	Year		Day	Month	Year

45. a) Exanthema ☐

b) Date of onset

Day	Month	Year		Day	Month	Year

46. a) Pruritus ☐

b) Date of onset

Day	Month	Year		Day	Month	Year

47. a) Numbness in the extremities ☐

b) Date of onset

Day	Month	Year

Day	Month	Year

48. a) Edema ☐

b) Date of onset

Day	Month	Year

Day	Month	Year

Site ID: ☐ Unique participant ID: ☐☐☐☐

49. a) Any medicine for this infection up to now? ☐

b) If yes, please, give details ______________________________

____

EXAMINATION

1 = No 2 = Yes

50. Capillary refill ☐
51. Filiform pulse ☐
52. Cold extremities ☐
53. Hypotension/shock ☐
54. Dyspnoea ☐
55. Dehydration ☐
56. Pale skin/mucosa ☐
57. Edema ☐
58. Jaundice ☐
59. Oropharyngeal hyperemia ☐

60. a) Lynphadenopathy ☐ e) Supraclavicular ☐

b) Cervical ☐ f) Axilar ☐

c) Retro auricular ☐ g) Epitroclear ☐

d) Occipital ☐ h) Inguinal ☐

Site ID: ☐ Unique participant ID: ☐☐☐☐

61. a) Exanthema ☐ e) Erithema ☐

b) Macular ☐ f) Enanthema ☐

c) Maculo-papular ☐ g) Other ☐

d) Vesiculosus ☐ ______________________

62. Haemorrhage ☐

63. Petechia ☐

64. Purpura ☐

65. Gum bleeding ☐

66. Epistaxe ☐

67. a) Cardiac abnormalities ☐ d) Coarse crackles ☐

b) Murmur ☐ e) Wheezes ☐

c) Fine crackles ☐

68. a) Abdomial abnormalities ☐ d) Hepatomegaly ☐

b) Pain on palpation ☐ e) Splenomegaly ☐

c) Ascitis ☐

69. Neurological abnormalities ☐

b) Lethargy ☐

c) Agitation ☐

d) Other ☐

Please, specify ____________________

70. a) Rheumatological abnormalities ☐

b) Edema ☐

c) Other ☐ Please, specify
