# Supplementary material for: Study on the persistence of Zika virus (ZIKV) in body fluids of patients with ZIKV infection in Brazil
Source: BMC Infect Dis. 2018 Jan 22;18:49. doi: 10.1186/s12879-018-2965-4 (PMC5778641; doi:10.1186/s12879-018-2965-4)
Supplement: Supplementary file 7 — Consultation_form_2st_visit. (PDF 350 kb) [file 12879_2018_2965_MOESM7_ESM.pdf]

Symptoms/Signs version 1.1 12-Sep-16	Persistence of Zika virus in body fluids of patients with Zika virus infection Consultation form - 2nd consult	CON2 Page 1/8 v1.1(12/09/2016

Site ID: ☐ Unique participant ID: ☐☐☐☐

(1 = Rio, 2 = Manaus, 3 = Recife)

Participant type: ☐ GAL number: ☐☐☐☐☐☐☐☐☐☐☐

(1 = Index, 2 = Contact symptomatic, 3 = Contact asymptomatic)

If household contact/sexual partner, number of Index: ☐☐☐☐

VITAL SIGNS

1. Date of second appointment

Day	Month	Year

2. a) Highest temperature measured so far °C ☐☐, ☐

b) Current temperature °C ☐☐, ☐

c) Date of the end of fever

Day	Month	Year

3. Heart rate per minute ☐☐☐

4. Respiratory rate per minute ☐☐

5. a) Blood pressure sitted (Systolic/Dyastolic) mmHg ☐☐☐ / ☐☐☐

b) Bood pressute lying down (Systolic/Dyastolic) mmHg ☐☐☐ / ☐☐☐

6. Weight Kg ☐☐☐, ☐

7. Height m ☐, ☐☐

SYMPTOMS

1 = No 2 = Yes

8. a) Chill ☐

b) Date of onset

Day	Month	Year

c) Date of end

Day	Month	Year

9. a) Headache ☐

b) Date of onset

Day	Month	Year

c) Date of end

Day	Month	Year

Site ID: ☐ Unique participant ID: ☐☐☐☐

10. a) Photophobia ☐

b) Date of onset Day | Month | Year c) Date of end Day | Month | Year

11. a) Retro orbital pain ☐

b) Date of onset Day | Month | Year c) Date of end Day | Month | Year

12. a) Ocular burning ☐

b) Date of onset Day | Month | Year c) Date of end Day | Month | Year

13. a) Oropheryngeal pain ☐

b) Date of onset Day | Month | Year c) Date of end Day | Month | Year

14. a) Horseness ☐

b) Date of onset Day | Month | Year c) Date of end Day | Month | Year

15. a) Change in taste ☐

b) Date of onset Day | Month | Year c) Date of end Day | Month | Year

16. a) Earache ☐

b) Date of onset Day | Month | Year c) Date of end Day | Month | Year

17. a) Coryza ☐

b) Date of onset Day | Month | Year c) Date of end Day | Month | Year

Site ID: ☐ Unique participant ID: ☐☐☐☐

18. a) Nasal congestion ☐

b) Date of onset

Day		Month		Year			

c) Date of end

Day		Month		Year			

19. a) Sudoresis ☐

b) Date of onset

Day		Month		Year			

c) Date of end

Day		Month		Year			

20. a) Prostration ☐

b) Date of onset

Day		Month		Year			

c) Date of end

Day		Month		Year			

21. a) Myalgia ☐

b) Date of onset

Day		Month		Year			

c) Date of end

Day		Month		Year			

22. a) Lombalgia ☐

b) Date of onset

Day		Month		Year			

c) Date of end

Day		Month		Year			

23. a) Muscular weakness ☐

b) Date of onset

Day		Month		Year			

c) Date of end

Day		Month		Year			

24. a) Arthralgia ☐

b) Date of onset

Day		Month		Year			

c) Date of end

Day		Month		Year			

Site ID: ☐ Unique participant ID: ☐☐☐☐

d) Site of arthalgia

1. Shoulder ☐
2. Elbow ☐
3. Knee ☐
4. hip ☐
5. Ankle ☐

5. Small articulations ☐
6. Symetric ☐
7. Migratory ☐

25. a) Anorexia ☐

b) Date of onset

Day		Month		Year			

c) Date of end

Day		Month		Year			

26. a) Nausea ☐

b) Date of onset

Day		Month		Year			

c) Date of end

Day		Month		Year			

27. a) Vomit ☐

b) Date of onset

Day		Month		Year			

c) Date of end

Day		Month		Year			

28. a) Abdominal pain ☐

b) Date of onset

Day		Month		Year			

c) Date of end

Day		Month		Year			

29. a) Choluria ☐

b) Date of onset

Day		Month		Year			

c) Date of end

Day		Month		Year			

30. a) Dysuria ☐

b) Date of onset

Day		Month		Year			

c) Date of end

Day		Month		Year			

Symptoms/Signs version 1.1 12-Sep-16	Persistence of Zika virus in body fluids of patients with Zika virus infection Consultation form - 2nd consult	CON2 Page 5/8 v1.1(12/09/2016

Site ID: ☐ Unique participant ID: ☐☐☐☐

31. a) Dyarrhoea ☐

b) Date of onset | Day | Month | Year | c) Date of end | Day | Month | Year |

32. a) Dyspnoea ☐

b) Date of onset | Day | Month | Year | c) Date of end | Day | Month | Year |

33. a) Cough ☐

b) Date of onset | Day | Month | Year | c) Date of end | Day | Month | Year |

34. a) Haemarrhage ☐

b) Date of onset | Day | Month | Year | c) Date of end | Day | Month | Year |

35. a) Epistaxe ☐

b) Date of onset | Day | Month | Year | c) Date of end | Day | Month | Year |

36. a) Gum bleeding ☐

b) Date of onset | Day | Month | Year | c) Date of end | Day | Month | Year |

37. a) Metrorrhagia ☐

b) Date of onset | Day | Month | Year | c) Date of end | Day | Month | Year |

38. a) Hematuria ☐

b) Date of onset | Day | Month | Year | c) Date of end | Day | Month | Year |

Site ID: ☐ Unique participant ID: ☐☐☐☐

39. a) Hematemesis ☐

b) Date of onset

Day		Month		Year			

c) Date of end

Day		Month		Year			

40. a) Melena ☐

b) Date of onset

Day		Month		Year			

c) Date of end

Day		Month		Year			

41. a) Hemoptysis ☐

b) Date of onset

Day		Month		Year			

c) Date of end

Day		Month		Year			

42. a) Erythema ☐

b) Date of onset

Day		Month		Year			

c) Date of end

Day		Month		Year			

43. a) Exanthema ☐

b) Date of onset

Day		Month		Year			

c) Date of end

Day		Month		Year			

44. a) Pruritus ☐

b) Date of onset

Day		Month		Year			

c) Date of end

Day		Month		Year			

45. a) Numbness in the extremities ☐

b) Date of onset

Day		Month		Year			

c) Date of end

Day		Month		Year			

46. a) Edema ☐

b) Date of onset

Day		Month		Year			

c) Date of end

Day		Month		Year			

Site ID: ☐ Unique participant ID: ☐☐☐☐

47. a) Any medicine for this infection up to now? ☐

b) If yes, please, give details ______________________

EXAMINATION

1 = No (***Skip to the next***) 2 = Yes

48. Capillary refill ☐

49. Filiform pulse ☐

50. Cold extremities ☐

51. Hypotension/shock ☐

52. Dyspnoea ☐

53. Dehydration ☐

54. Pale skin/mucosa ☐

55. Edema ☐

56. Jaundice ☐

57. Oropharyngeal hyperemia ☐

58. a) Lynphadenopathy ☐

b) Cervical ☐

c) Retro auricular ☐

d) Occipital ☐

e) Supraclavicular ☐

f) Axilar ☐

g) Epitroclear ☐

h) Inguinal ☐

59. a) Exanthema ☐

b) Macular ☐

c) Maculo-papular ☐

d) Vesiculosus ☐

e) Erythema ☐

f) Enanthema ☐

g) Other ☐

Site ID: ☐ Unique participant ID: ☐☐☐☐

60. Haemorrhage ☐

61. Petechia ☐

62. Purpura ☐

63. Gum bleeding ☐

64. Epistaxe ☐

65. a) Cardiac abnormalities ☐
 b) Murmur ☐
 c) Fine crackles ☐
 d) Coarse crackles ☐
 e) Wheezes ☐

66. a) Abdomial abnormalities ☐
 b) Pain on palpation ☐
 c) Ascitis ☐
 d) Hepatomegaly ☐
 e) Splenomegaly ☐

67. Neurological abnormalities ☐
 b) Lethargy ☐
 c) Agitation ☐
 d) Other ☐
 Please, specify ____________________

68. a) Rheumatological abnormalities ☐
 b) Edema ☐
 c) Other ☐
 Please, specify ____________________
